# Improvement of High-Throughput Experimentation Using Synthesis Robots by the Implementation of Tailor-Made Sensors

**DOI:** 10.3390/polym14030361

**Published:** 2022-01-18

**Authors:** Timo Schuett, Manuel Wejner, Julian Kimmig, Stefan Zechel, Timm Wilke, Ulrich S. Schubert

**Affiliations:** 1 Laboratory of Organic and Macromolecular Chemistry (IOMC), Friedrich Schiller University Jena, Humboldtstr. 10, 07743 Jena, Germany; timo.schuett@uni-jena.de (T.S.); julian.kimmig@uni-jena.de (J.K.); stefan.zechel@uni-jena.de (S.Z.); 2 Jena Center of Soft Matter (JCSM), Friedrich Schiller University Jena, Philosophenweg 7, 07743 Jena, Germany; manuel.wejner@uni-jena.de; 3 Institute for Inorganic Chemistry and Analytical Chemistry, Chemistry Education, Friedrich Schiller University Jena, August-Bebel-Strasse 2, 07743 Jena, Germany

**Keywords:** automation, self-produced sensor, reaction monitoring, high-throughput experimentation, online characterization, UV reaction

## Abstract

A small, low-cost, self-produced photometer is implemented into a synthesis robot and combined with a modified UV chamber to enable automated sampling and online characterization. In order to show the usability of the new approach, two different reversible addition–fragmentation chain transfer (RAFT) polymers were irradiated with UV light. Automated sampling and subsequent characterization revealed different reaction kinetics depending on polymer type. Thus, a long initiation time (20 min) is required for the end-group degradation of poly(ethylene glycol) ether methyl methacrylate (poly(PEGMEMA)), whereas poly(methyl methacrylate) (PMMA) is immediately converted. Lastly, all photometric samples are characterized via size-exclusion chromatography using UV and RI detectors to prove the results of the self-produced sensor and to investigate the molar mass shift during the reaction.

## 1. Introduction

The ongoing digitalization in research, e.g., in chemistry, led to a significant need for experimental data and a higher throughput of reactions, which can be realized using automated, parallelized, and miniaturized synthesizers [1,2], and fast and accurate characterization connected to the respective device [3]. Consequently, a simple and rapid optimization of reaction conditions [4], screening of catalyst efficiencies [5], and investigation of nanoparticle properties [6,7] can be realized using such approaches. Siegwart and coworkers performed 1536 nanoparticle formulations for intracellular delivery [6]. To analyze this large number of samples, characterization devices connected to the automated synthesizer are required that enable the automated online reaction monitoring [8,9,10]. Thus, reactions are performed almost autonomously, while the synthesis progress and measurement data can be monitored at any time and from any location. Those approaches are also known from flow-chemistry experimentation [11]. However, synthetic robots feature only limited free available space inside, and the size of the characterization device is consequently essential. Devices larger than the automated synthesizer need an injection valve [3] or an autosampler aligned to the synthesis robot [12], which significantly complicates online characterization.

Consequently, the current study focusses on the implementation of a small, low-cost self-produced sensor into a synthesis robot enabling automated online characterization. Therefore, we used prior developed digital measuring system *LabPi* [13,14]. This system consists of a single-board computer (Raspberry Pi), a tailor-made adapter board, and various small and individually arrangeable sensor modules (e.g., temperature, pressure, pH value, gas sensor, conductivity, and a photometer). The computer is connected to a touch display that can be utilized to operate the analytical software [13,14]. This system is applicable in, for instance, reaction optimization by in situ measurements of pH value and temperature [15], air-quality tests by measuring CO_2_ concentration [16], hydrogen detection during photocatalytic water splitting [17], and general chemistry education [13,14,18], for which it was originally developed.

In order to show the benefit of the combination of low-cost and small sensors with a synthesis robot, we utilized the photoinduced cleavage of a reversible addition–fragmentation chain transfer (RAFT) end group as a model reaction. The RAFT reaction [19] as controlled radical polymerization is one of the most frequently utilized reaction processes to achieve low-dispersity polymers since this technique is applicable for a wide range of different monomers, such as methacrylates [20], acrylates [4], acrylamides [21,22], styrenes [23], or vinyl ethers [24]. However, due to the sulfurous end group (dithioester, trithiocarbonate, dithiocarbamate, or xanthate [24]), the resulting polymer is colored and sometimes smells, which makes the polymers unsuitable for several applications [25]. For further use of such low-dispersity polymers, they must undergo end-group modification such as a hetero-Diels–Alder reaction [26] or reaction with an amino moiety [27,28]. An additional procedure for postpolymerization modification is the degradation of the end group by irradiation with UV light [29]. After such an end-group modification, these polymers can be applied, e.g., for optoelectronic applications [30] or pharmaceutical purposes such as drug [31] or gene delivery [32].

The approach presented here utilizes a modified UV chamber for automated sampling by a synthesis robot to enable automated reaction monitoring by the implemented LabPi system. Consequently, our approach can be utilized to automatically design RAFT-based polymers without the typical end group for various applications.

Lastly, several RAFT end-group degradation reactions as postpolymerization modification were performed and automatically characterized to verify the new system.

## 2. Materials and Methods

### 2.1. Materials and Methods

All chemicals were used as received from Sigma Aldrich (Merck KGaA, Darmstadt, Germany) (azobisisobutyronitrile (AIBN), 2-cyano-2-propylbenzodithioat (CPDB), methyl methacrylate (MMA), poly(ethylene glycol) ether metyl methacrylate (poly (PEGMEMA)) (M_n_ = 500 g mol^−1^)), VWR (Darmstadt, Germany) (tetrahydrofuran (THF)) and Acros Organics (Thermo Fisher Scientific, Schwerte, Germany) (dimethylformamide (DMF)), if not otherwise stated. The DMF solvent was dried over a molecular sieve and stored under a nitrogen atmosphere. Liquid monomer PEGMEMA that was used was destabilized over a short AlOx column (neutral AlOx, obtained from Molecula (Munich, Germany)). Dialysis tubing was purchased from Spectrum Labs^TM^ (Ravensburg, Germany) (Spectra/PorTM, prewetted tubing, 3.5 kDa) and rinsed with the respective solvent before use.

UV reactions were performed utilizing a UVACUBE 100 from Dr. Hoenle AG (Gilching, Germany) equipped with a mercury lamp.

The 3D printing was executed using a Prusa i3 MK3S (Prague, Czech Republic) utilizing filament purchased from 3D Prima: polylactic acid (PLA) and polyethylene terephthalate modified with glycol (PETG).

The photometer was equipped with a light-emitting diode (LED) from Everlight (Karlsruhe, Germany) (16–213/BHC-AN1P2/3T), emitting light at a wavelength of 468 nm (blue LED), a sensor from AMS (TSL2561T), and a semimicro-quartz glass cuvette from Rotilabo with a maximal volume of 1.4 mL. The photometer was connected to a Raspberry Pi 3 model B+ utilizing Raspberry Pi OS (Linux 10 “buster”, Raspberry Pi Foundation, Cambridge, United Kingdom) and LabPi software (Version 0.23, Friedrich Schiller University Jena, Germany) [33] to access the photometer. Moreover, the photometer was equipped with a semimicro-cuvette from ROTILABO composed of quartz glass with a maximal volume of 1.4 mL.

Automated liquid transfers were performed using a Chemspeed SWING XL (Basel, Swiss) automated parallel synthesizer equipped with an overhead robot arm that could pick up several tools such as a 4-needle head (4-NH) for automated liquid handling. The 4-NH is equipped with septum piercing needles.

Nuclear magnetic resonance spectra were measured using a Bruker AC 300 (Billerica, MA, USA) (300 MHz) spectrometer at 298 K. The chemical shift is given in parts per million (ppm on the *δ* scale) related to deuterated solvent.

Size-exclusion chromatography measurements (SEC) were performed with the following setup: Shimadzu (Kyōto, Japan) with CBM-20A (system controller), DGU-14A (degasser), LC-20AD (pump), SIL-20AHT (autosampler), CTO-10AC vp (oven), SPD-20A (UV detector), RID-10A (RI detector), PSS SDV guard/1000 Å/1,000,000 Å (5 μm particle size, supplier: PSS GmbH (Mainz, Germany), separation range: 400 to 1,000,000 g mol^−1^) chloroform/isopropanol/triethyl-amine [94/2/4] with 1 mL min^−1^ at 40 °C, PEG and PMMA standards (details for the standards are listed in the Supplementary Materials).

### 2.2. Polymer Synthesis 

**Scheme 1:**
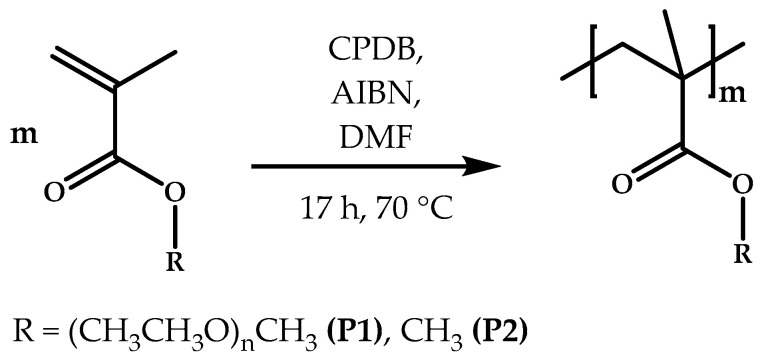
Schematic representation of synthesis of **P1** and **P2**.

A schematic representation of the synthesis of the two polymers **P1** and **P2** is shown in Scheme 1:

Solutions of the initiator (AIBN), chain-transfer agent (CPDB), and monomer (PEGMEMA (**P1**) or PMMA (**P2**)) in DMF were prepared with a [M]:[CTA]:[I] ratio of 50:1:0.25 (**P1**) and 200:1:0.25 (**P2**) in a round bottom flask. After closing the reaction vessel with a septum, the reaction mixture was degassed by flushing with nitrogen for 30 min. Solution polymerizations were carried out in a preheated oil bath at 70 °C for 17 h. All amounts and volumes are listed in Table 1. The respective polymers (PMMA, 53.7 g; poly(PEGMEMA), 49.2 g) were obtained after dialysis in THF (5 × 600 mL, solvent exchange after 12 h) and after drying in vacuo.

**P1**: ^1^H NMR (300 MHz, CDCl_3_, *δ*): 0.51–1.11 (m, 3H), 1.55–2.16 (m, 2H), 3.43–3.79 (m, 38H) ppm.

SEC (chloroform/isopropanol/triethylamine [94/2/4], PEG standard): M_n_ = 11,900 g mol^−1^, M_w_ = 14,700 g mol^−1^, Ð = 1.20.

SEC (chloroform/isopropanol/triethylamine [94/2/4], PMMA standard): M_n_ = 22,600 g mol^−1^, M_w_ = 22,300 g mol^−1^, Ð = 1.15.

**P2**: ^1^H NMR (300 MHz, CDCl_3_, *δ*): 0.57–1.04 (m, 3H), 1.55–2.04 (m, 3H), 3.35–3.78 (m, 4H) ppm.

SEC (chloroform/isopropanol/triethylamine [94/2/4], PMMA standard): M_n_ = 19,500 g mol^−1^, M_w_ = 21,200 g mol^−1^, Ð = 1.17.

### 2.3. UV-Induced RAFT End-Group Degradation

Every one of the following liquid transfers was automatically performed by utilizing an automated synthesizer if not otherwise stated.

First, 2 g of the respective polymer was dissolved in 15 mL THF and filled in a 20 mL headspace vial, equipped with a slotted septum, and manually placed inside the UVA-CUBE. The semimicro-cuvette placed inside the photometer was filled with pure THF to measure the reference point. Afterwards, the THF was removed, disposed of, and 1 mL of the polymeric solution was transferred into the cuvette to measure the extinction of the sample before UV irradiation. Subsequently, 0.4 mL of the polymeric solution in the cuvette was transferred into an SEC vial and filled up with 0.6 mL SEC solvent (chloroform/isopropanol/triethylamine (94/2/4)). Afterwards, the cuvette was rinsed four times with THF. The UV irradiation of the polymeric solution was started, and the reaction was monitored by taking a sample every ten minutes for a total of 100 min in the same route as mentioned before, including SEC sampling and rinsing. Lastly, polymers **P3** (obtained from **P1**) and **P4** (obtained from **P2**) were prepared and investigated via NMR and SEC after solvent evaporation.

**P3**: ^1^H NMR (300 MHz, CDCl_3_, *δ*): 0.55–1.11 (m, 3H), 1.51–2.10 (m, 2H), 3.44–3.79 (m, 37H) ppm.

SEC (chloroform/isopropanol/triethylamine [94/2/4], PEG standard): M_n_ = 11,300 g mol^−1^, M_w_ = 13,600 g mol^−1^, Ð = 1.21.

SEC (chloroform/isopropanol/triethylamine [94/2/4], PMMA standard): M_n_ = 22,600 g mol^−1^, M_w_ = 26,800 g mol^−1^, Ð = 1.19.

**P4**: ^1^H NMR (300 MHz, CDCl_3_, *δ*): 0.56–1.12 (m, 3H), 1.57–2.13 (m, 2H), 3.39–3.62 (m, 3H) ppm.

SEC (chloroform/isopropanol/triethylamine [94/2/4], PMMA standard): M_n_ = 17,500 g mol^−1^, M_w_ = 20,700 g mol^−1^, Ð = 1.18.

## 3. Results and Discussion

**Figure 1:**
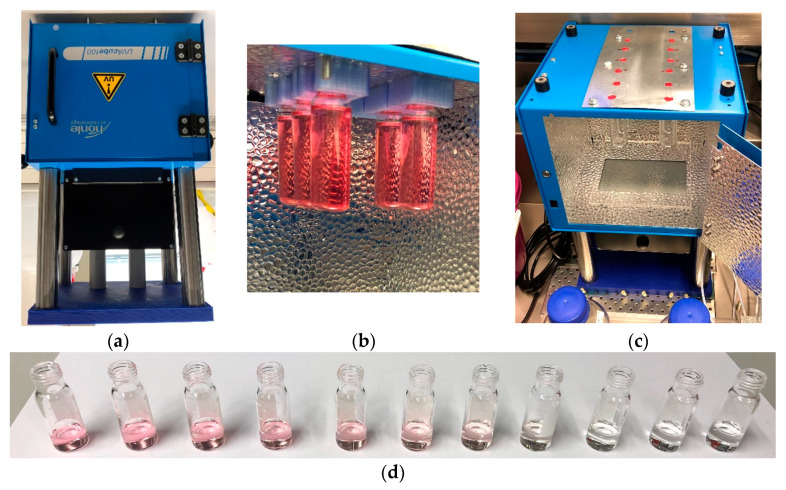
Modified UV chamber and experiments: (**a**) Front view of UV chamber; (**b**) view inside UV chamber with for wells mounted on top filled with **P1** dissolved in THF; (**c**) top view of UV chamber implemented in synthesis robot; and (**d**) photometric samples of **E1** from (**left**) 0 min to (**right**) 100 min in 10 min intervals.

First, the UV chamber was modified in order to achieve compatibility with the synthesis robot and for automated sampling. Pictures of the modified UV chamber are shown in Figure 1. For this purpose, the commercially available chamber was turned upside down to enable both the irradiation of the reaction solution from the bottom of the chamber and automated sampling. To prevent the mercury lamp from overheating and to adjust the height, the whole chamber, including UV lamp, was mounted onto a pedestal (3D-printed base (PLA) and aluminum pillars, Figure 1a). Second, six holders suitable for the utilized headspace vials were 3D-printed (PETG) and fixed from the inside at the top of the chamber (Figure 1b). Both 3D models can be found in the supplementary materials. Lastly, to enable automated sampling by the synthesis robot, several holes were drilled into the top of the UV chamber, and a septum was fixed onto each using an aluminum plate (Figure 1c) to protect the environment from the UV light.

Furthermore, the photometer was placed inside the synthesis robot, and a hole was drilled through the cap of the measuring chamber and equipped with a septum to ensure the absence of environmental light inside.

For all UV experiments, poly(PEGMEMA) (**P1**) and PMMA (**P2**) were utilized since they are commonly used polymers [34]. Consequently, the working principle and effectiveness could be studied in detail. Both polymers were synthesized by a RAFT polymerization using the standard conditions established in the Schubert group [35].

As a first experiment, poly(PEGMEMA) (**P1**) was diluted in THF and irradiated with UV light. This experiment was repeated four times (**E1–E5**). Afterwards, the same experiment was performed five times with a solution of PMMA (**P2**) in THF (**E6–E10**). The color within the reaction changed from pink to colorless (Figure 1d).

**Figure 2:**
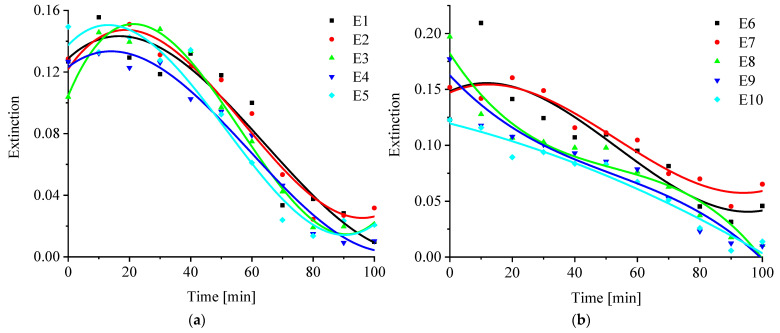
Diagram of photometric measurements of UV-induced degradation of (**a**) poly(PEGMEMA) (**P1**, **E1–E5**) and (**b**) PMMA (**P2**, **E6–E10**). Samples were taken every 10 min for a period of 100 min. Each experiment was fitted utilizing a cubic polynomial fit.

Furthermore, these experiments were analyzed via photometric measurements. The LabPi photometer enables the acquisition of light intensities via different LEDs and a light intensity sensor (AMS TSL2651T). Intensity values are transmitted to the measuring station to determine the absorbance and are displayed in a live view on the touch display of the measuring station. As before, the cuvette was filled with THF as reference solution, and the reference value was recorded at a wavelength of 468 nm (blue LED), which was chosen due to the typical absorption of RAFT polymers synthesized using a dithiobenzoate-based RAFT agent [27]. Subsequently, absorbances of the analytical solutions were determined every 10 min over a period of 100 min. Between each measurement, the cuvette was automatically rinsed four times with THF before being filled with 1 mL of the reaction solution by the synthesis robot. After the measurement had finished, the reaction solution was transferred into an SEC vial, and recorded absorbances were exported for additional analysis. The corresponding diagram of absorbance as a function of time is shown in Figure 2.

Experiments **E1–E5** (Figure 2a) showed slightly ascending extinction within the first 20 min. Afterwards, signal intensity mostly linearly decreased for about 60 min and approximated the lower limit at the end of the experiment.

In contrast, experiments **E6–E10** (Figure 2b) revealed no ascending behavior in the beginning. Extinction mostly linearly decreased until it reached a limit after 90 min of reaction. Consequently, initiation time was significantly lower compared to the first experiments utilizing poly(PEGMEMA). However, the end of the reaction was also reached after 90 min, which indicated a lower reaction speed during the linear phase.

In order to prove the results of the self-produced sensor, every photometric sample was characterized utilizing size-exclusion chromatography (SEC) using a UV (UV lamp emitting mainly at 270 nm) and an RI detector, respectively. The UV results of **E1** and **E8** are shown in Figure 3. Diagrams of all UV spectra are represented in Supplementary Figures S3–S12.

**Figure 3:**
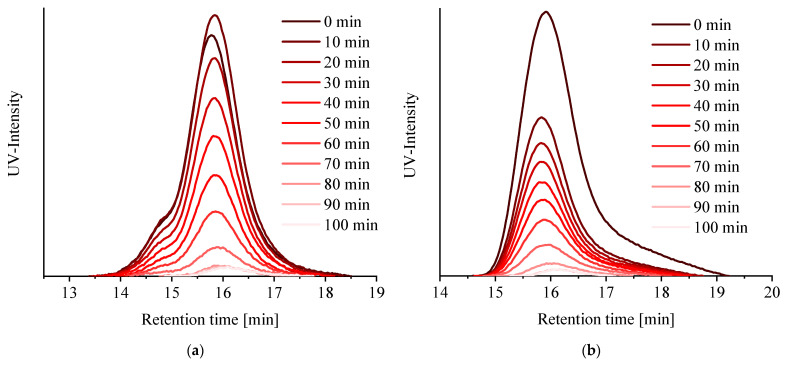
SEC curves of UV-induced degradation of (**a**) poly(PEGMEMA) (**P1**, **E1**) and (**b**) PMMA (**P2**, **E8**). Samples were taken every 10 min for a period of 100 min (chloroform/isopropanol/triethylamine (94/2/4); (**a**) PEG standard and (**b**) PMMA standard).

In both diagrams (Figure 3a, **E1**; Figure 3b, **E8**), the peak maximum remained nearly at the same retention time, while intensity decreased until the lowest maximum between the experiments (90 min) was reached. Consequently, the molar mass of the respective polymer did not change significantly during the reaction, whereas color changed continuously towards smaller extinction. These results prove the findings of the photometric experiments.

The resulting diagrams of the RI detector are represented in Supplementary Figures S13–S22. Compared to the UV detector, the RI detector resulted in higher signal intensity in the later samples, which were important to identify a potential molar mass shift due to fragmentation of the RAFT group during the reaction. Furthermore, the molar mass remained constant in the range of the measurement accuracy of the SEC during the experiment, as expected. A shift of the molar mass due to the potential degradation of the RAFT end group was not observed. Consequently, UV irradiation interacted with the labile RAFT end group, which led to the detected color change; however, it did not interact with the polymer chain, which was detected utilizing the RI detector.

## 4. Conclusions

Within this study, a new approach for an automated UV reaction connected to online photometric characterization within a synthetic robot was presented. For this purpose, a UV chamber and a self-produced photometer were implemented into a synthesis robot, both accessible by the synthesizer, enabling the automated sampling and subsequent online characterization of a UV-induced RAFT end-group degradation reaction. Within this context, two polymer types (poly(ethylene glycol) ether methyl methacrylate (poly(PEGMEMA) (**P1**) and poly(methyl methacrylate) (PMMA) (**P2**)) were investigated, each in five iterations, resulting in a color change from pink to colorless within 100 min of reaction time. This color change was reliably detected by the self-produced photometer and proofed by a UV detector had been coupled to a size-exclusion chromatography (SEC) machine. Interestingly, poly(PEGMEMA) revealed an initiation time of 20 min for the start of the reaction, followed by primarily linear behavior until all RAFT end groups had been converted. In contrast to this, PMMA showed linear behavior from the beginning until the end of the reaction.

Furthermore, a second detector coupled to the same SEC was applied to detect potential changes in the molar mass of the polymer. Consequently, the polymer chain itself did not interact with UV irradiation, and no coupling reactions were observed, whereas the RAFT end group showed cleavage and degradation.

Overall, the new setup enables a low-cost system for online photometric analysis and the possibility for high-throughput experimentation by increasing the number of photometers in the synthesis robot and reactors in the UV chamber. This approach can thus be the basis for high-throughput synthesis combined with fast and reliable online characterization, e.g., through the LabPi cloud platform COMPare [13,14,36]. Future work will aim to implement more devices to achieve parallelization of the experimentation and utilize other self-produced sensors.

## Figures and Tables

**Table 1:** Summary of utilized amounts and volumes for the RAFT polymerizations of MMA.

Polymer	Monomer	m(Monomer) (g)	V(DMF) (mL)	m(CPDB) (mg)	m(AIBN) (mg)
**P1**	PEGMEMA	100	100	885.4	164.2
**P2**	MMA	80	400	884.3	164.0

## Data Availability

Data presented in this study are available on request from the corresponding author.

## Supplementary Materials

The following are available online at https://www.mdpi.com/article/10.3390/polym14030361/s1: 3D model a: UV chamber pedestal base, 3D model b: vial holder; Table S1: Step-by-step protocol for the volumetric transfers of the reaction monitoring in the automated platform. The steps seven and eight were repeated every 10 min (in total eleven samples) until a reaction time of 100 min. Figure S1: 3D model of the podium printed as base for the UV reactor. Figure S2: 3D Model of the vial holder. Figure S3: Photo of the LabPi photometer, implemented into the synthesis robot. Left: Closed (during process). Right: Open (during maintenance). Figure S4: ^1^H NMR spectrum of polymer **P1** (300 MHz, CDCl_3_). Figure S5: ^1^H NMR spectrum of polymer **P2** (300 MHz, CDCl_3_). Figure S6: ^1^H NMR spectrum of polymer **P3** (300 MHz, CDCl_3_). Figure S7: ^1^H NMR spectrum of polymer **P4** (300 MHz, CDCl_3_). Figure S8: SEC curves of the UV-induced degradation of poly(PEGMEMA) (**P1**, **E1**). Samples were taken every ten minutes for a period of 100 min (chloroform/isopropanol/triethylamine [94/2/4], PEG and PMMA standard, UV detector (270 nm)). Figure S9: SEC curves of the UV-induced degradation of poly(PEGMEMA) (**P1**, **E1**). Samples were taken every ten minutes for a period of 100 min (chloroform/isopropanol/triethylamine [94/2/4], PEG- and PMMA-standard, RI detector). Figure S10: SEC curves of the UV-induced degradation of poly(PEGMEMA) (**P1**, **E2**). Samples were taken every ten minutes for a period of 100 min (chloroform/isopropanol/triethylamine [94/2/4], PEG and PMMA standard, UV detector (270 nm)). Figure S11: SEC curves of the UV-induced degradation of poly(PEGMEMA) (**P1**, **E2**). Samples were taken every ten minutes for a period of 100 min (chloroform/isopropanol/triethylamine [94/2/4], PEG and PMMA standard, RI detector). Figure S12: SEC curves of the UVinduced degradation of poly(PEGMEMA) (**P1**, **E3**). Samples were taken every ten minutes for a period of 100 min (chloroform/isopropanol/triethylamine [94/2/4], PEG and PMMA standard, UV detector (270 nm)). Figure S13: SEC curves of the UV-induced degradation of poly(PEGMEMA) (**P1**, **E3**). Samples were taken every ten minutes for a period of 100 min (chloroform/isopropanol/triethylamine [94/2/4], PEG and PMMA standard, RI detector). Figure S14: SEC curves of the UV-induced degradation of poly(PEGMEMA) (**P1**, **E4**). Samples were taken every ten minutes for a period of 100 min (chloroform/isopropanol/triethylamine [94/2/4], PEG and PMMA standard, UV detector (270 nm)). Figure S15: SEC curves of the UV-induced degradation of poly(PEGMEMA) (**P1**, **E4**). Samples were taken every ten minutes for a period of 100 min (chloroform/isopropanol/triethylamine [94/2/4], PEG and PMMA standard, RI detector). Figure S16: SEC curves of the UV-induced degradation of poly(PEGMEMA) (**P1**, **E5**). Samples were taken every ten minutes for a period of 100 min (chloroform/isopropanol/triethylamine [94/2/4], PEG and PMMA standard, UV-detector (270 nm)). Figure S17: SEC curves of the UV-induced degradation of poly(PEGMEMA) (**P1**, **E5**). Samples were taken every ten minutes for a period of 100 min (chloroform/isopropanol/triethylamine [94/2/4], PEG and PMMA standard, RI detector). Figure S18: SEC curves of the UV-induced degradation of PMMA (**P2**, **E6**). Samples were taken every ten minutes for a period of 100 min (chloroform/isopropanol/triethylamine [94/2/4], PMMA standard, UV detector (270 nm)). Figure S19: SEC curves of the UV-induced degradation of PMMA (**P2**, **E6**). Samples were taken every ten minutes for a period of 100 min (chloroform/isopropanol/triethylamine [94/2/4], PMMA standard, RI detector). Figure S20: SEC curves of the UV-induced degradation of PMMA (**P2**, **E7**). Samples were taken every ten minutes for a period of 100 min (chloroform/isopropanol/triethylamine [94/2/4], PMMA standard, UV detector (270 nm)). Figure S21: SEC curves of the UV-induced degradation of PMMA (**P2**, **E7**). Samples were taken every ten minutes for a period of 100 min (chloroform/isopropanol/triethylamine [94/2/4], PMMA standard, RI detector). Figure S22: SEC curves of the UV-induced degradation of PMMA (**P2**, **E8**). Samples were taken every ten minutes for a period of 100 min (chloroform/isopropanol/triethylamine [94/2/4], PMMA standard, UV detector (270 nm)). Figure S23: SEC curves of the UV-induced degradation of PMMA (**P2**, **E8**). Samples were taken every ten minutes for a period of 100 min (chloroform/isopropanol/triethylamine [94/2/4], PMMA standard, RI detector). Figure S24: SEC curves of the UV induced degradation of PMMA (**P2**, **E9**). Samples were taken every ten minutes for a period of 100 min (chloroform/isopropanol/triethylamine [94/2/4], PMMA standard, UV detector (270 nm)). Figure S25: SEC curves of the UV-induced degradation of PMMA (**P2**, **E9**). Samples were taken every ten minutes for a period of 100 min (chloroform/isopropanol/triethylamine [94/2/4], PMMA standard, RI detector). Figure S26: SEC curves of the UV-induced degradation of PMMA (**P2**, **E10**). Samples were taken every ten minutes for a period of 100 min (chloroform/isopropanol/triethylamine [94/2/4], PMMA standard, UV detector (270 nm)). Figure S27: SEC curves of the UV-induced degradation of PMMA (**P2**, **E10**). Samples were taken every ten minutes for a period of 100 min (chloroform/isopropanol/triethylamine [94/2/4], PMMA standard, RI detector). Table S2: Average molar mass and dispersity of each sampling within experiment **E1**. Values obtained by SEC measurements using a RI detector (chloroform/isopropanol/triethylamine [94/2/4], PEG standard). Table S3: Average molar mass and dispersity of each sampling within experiment **E1**. Values obtained by SEC measurements using an RI detector (chloroform/isopropanol/triethylamine [94/2/4], PMMA standard). Table S4: Average molar mass and dispersity of each sampling within experiment **E2**. Values obtained by SEC measurements using an RI detector (chloroform/isopropanol/triethylamine [94/2/4], PEG standard). Table S5: Average molar mass and dispersity of each sampling within experiment **E2**. Values obtained by SEC measurements using an RI detector (chloroform/isopropanol/triethylamine [94/2/4], PMMA standard). Table S6: Average molar mass and dispersity of each sampling within experiment **E3**. Values obtained by SEC measurements using an RI detector (chloroform/isopropanol/triethylamine [94/2/4], PEG standard). Table S7: Average molar mass and dispersity of each sampling within experiment **E3**. Values obtained by SEC measurements using a RI detector (chloroform/isopropanol/triethylamine [94/2/4], PMMA standard). Table S8: Average molar mass and dispersity of each sampling within experiment **E4**. Values obtained by SEC measurements using a RI detector (chloroform/isopropanol/triethylamine [94/2/4], PEG standard). Table S9: Average molar mass and dispersity of each sampling within experiment **E4**. Values obtained by SEC measurements using a RI detector (chloroform/isopropanol/triethylamine [94/2/4], PMMA standard). Table S10: Average molar mass and dispersity of each sampling within experiment **E5**. Values obtained by SEC measurements using a RI detector (chloroform/isopropanol/triethylamine [94/2/4], PEG standard). Table S11: Average molar mass and dispersity of each sampling within experiment **E5**. Values obtained by SEC measurements using an RI detector (chloroform/isopropanol/triethylamine [94/2/4], PMMA standard). Table S12: Average molar mass and dispersity of each sampling within experiment **E6**. Values obtained by SEC measurements using an RI detector (chloroform/isopropanol/triethylamine [94/2/4], PMMA standard). Table S13: Average molar mass and dispersity of each sampling within experiment **E7**. Values obtained by SEC measurements using an RI detector (chloroform/isopropanol/triethylamine [94/2/4], PMMA standard). Table S14: Average molar mass and dispersity of each sampling within experiment **E8**. Values obtained by SEC measurements using an RI detector (chloroform/isopropanol/triethylamine [94/2/4], PMMA standard). Table S15: Average molar mass and dispersity of each sampling within experiment **E9**. Values obtained by SEC measurements using a RI-detector (chloroform/isopropanol/triethylamine [94/2/4], PMMA standard). Table S16: Average molar mass and dispersity of each sampling within experiment **E10**. Values obtained by SEC measurements using a RI-detector (chloroform/isopropanol/triethylamine [94/2/4], PMMA standard). Table S17: Average values of the average molar mass, the respective standard deviation (SD) and dispersity of each sampling within the respective experiment type (**E1** to **E5** and **E6** to **E10**) Values obtained by SEC measurements using an RI detector (chloroform/isopropanol/triethylamine [94/2/4], PEG/PMMA standard).
