# Supplementary material for: Improvement of High-Throughput Experimentation Using Synthesis Robots by the Implementation of Tailor-Made Sensors
Source: Polymers (Basel). 2022 Jan 18;14(3):361. doi: 10.3390/polym14030361 (PMC8838243; doi:10.3390/polym14030361)
Supplement: Supplementary file 1 [file polymers-14-00361-s001.zip › polymers-1515106-Supplementary.pdf]

Supporting Information

Improvement of high-throughput experimentation using synthesis robots by the implementation of self-made sensors

Timo Schuett,[a, b] Manuel Wejner,[b, c] Julian Kimmig,[a, b] Stefan Zechel,[a, b] Timm Wilke[b,c,] * and Ulrich S. Schubert[a, b,] *

[a] Laboratory of Organic and Macromolecular Chemistry (IOMC), Friedrich Schiller University Jena, Humboldtstr. 10, 07743 Jena, Germany

[b] Jena Center for Soft Matter (JCSM), Friedrich Schiller University Jena, Philosophenweg 7, 07743 Jena, Germany

[c] Institute for Inorganic Chemistry and Analytical Chemistry, Didactics of Chemistry, Friedrich Schiller University Jena, August-Bebel-Strasse 2, 07743 Jena, Germany

Table of content

1. Step-by-step protocol RAFT-end group degradation 3

2. 3D-Models 4

3. LabPi measuring station 5

4. NMR spectroscopy 6

5. SEC-Diagrams 8

6. SEC-Data 18

1. **Step-by-step protocol RAFT-end group degradation**

Table S1: Step-by-step protocol for the volumetric transfers of the reaction monitoring in the automated platform. The steps seven and eight were repeated every 10 minutes (in total eleven samples) until a reaction time of 100 minutes.

Step		Task	Description
**1**		**Macrotask**	**Measurement of reference and time (t) = 0 min**
	1.1	Liquid transfer	THF (1 mL) to UV/Vis-sensor
	1.2	Show dialog	‘Measurement reference’
	1.3	Liquid transfer	1.3 mL from UV/Vis-sensor to waste
	1.4	Liquid transfer	1 mL from UV-reactor to UV-sensor
	1.5	Liquid transfer	1 mL from UV-sensor to UV-sensor (mixing)
	1.6	Show dialog	‘Measurement t = 0 min’
2		Show dialog	‘UV reactor on’
3		Set timer	Set timer ‘sampling’
4		Liquid transfer	1.3 mL from UV/Vis-sensor to waste
**5**		**Macrotask**	**Rinsing (loop, four times)**
	5.1	Liquid transfer	THF (1 mL) to UV-sensor
	5.2	Liquid transfer	1.3 mL from UV/Vis-sensor to waste
6		Wait	Waiting for 10 minutes after timer ‘sampling’
**7**		**Macrotask**	**Sampling t = 10 min**
	7.1	Liquid transfer	1 mL from UV-reactor to UV-sensor
	7.2	Liquid transfer	1 mL from UV-sensor to UV-sensor (mixing)
	7.3	Show dialog	‘Measurement t = 10 min’
	7.4	Liquid transfer	1.3 mL from UV/Vis-sensor to waste
**8**		**Macrotask**	**Rinsing (loop, four times)**
	8.1	Liquid transfer	THF (1 mL) to UV-sensor
	8.2	Liquid transfer	1.3 mL from UV/Vis-sensor to waste

2. 3D-Models

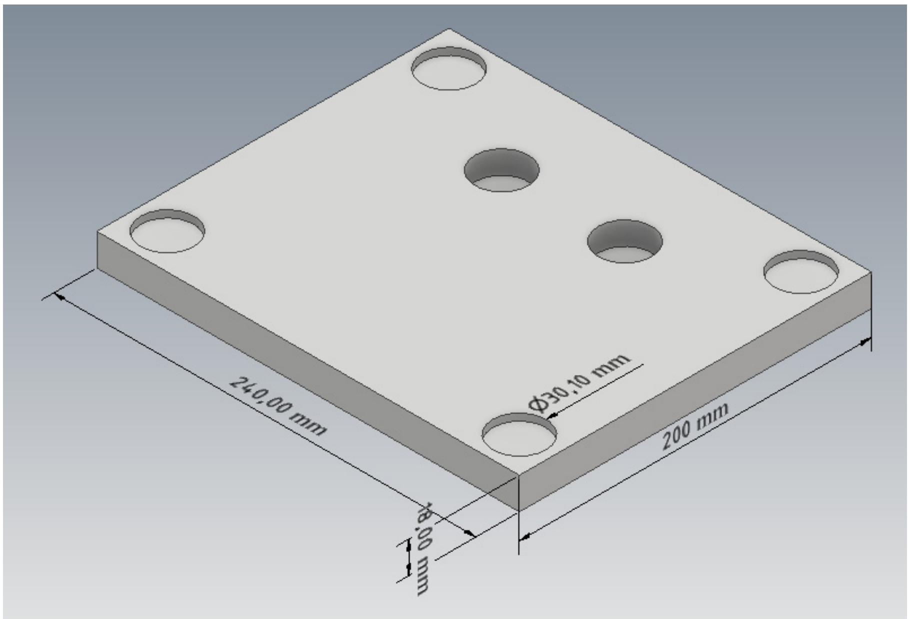

Figure S1: 3D-Model of the podium, printed as base for the UV-reactor.

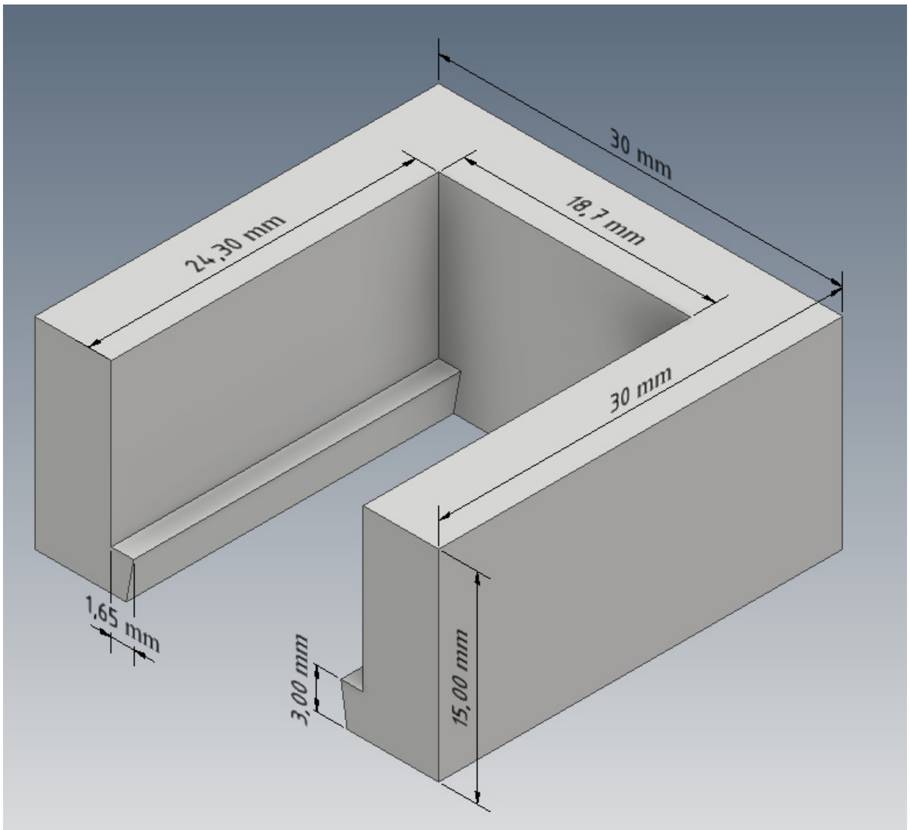

Figure S2: 3D-Model of the vial holder.

3. LabPi measuring station

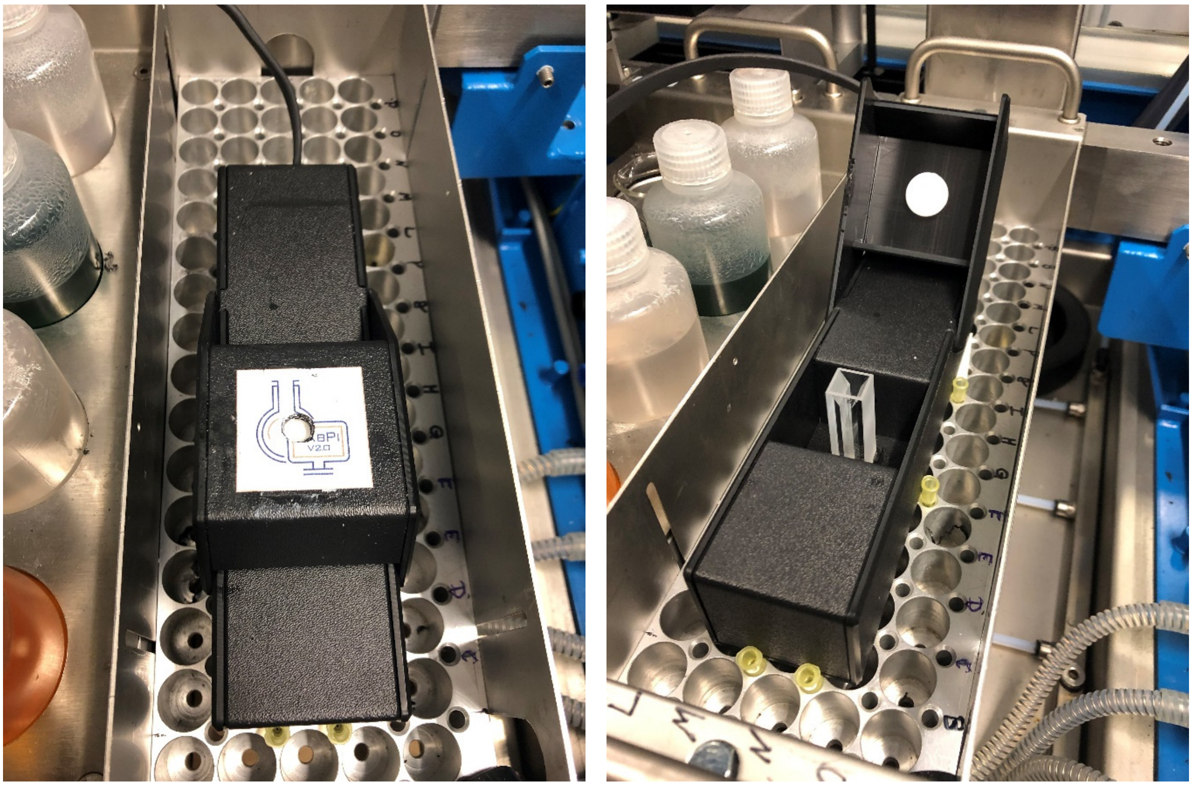

Figure S3: Photo of the LabPi photometer, implemented into the synthesis robot. Left: Closed (during process). Right: Open (during maintenance).

4. NMR spectroscopy

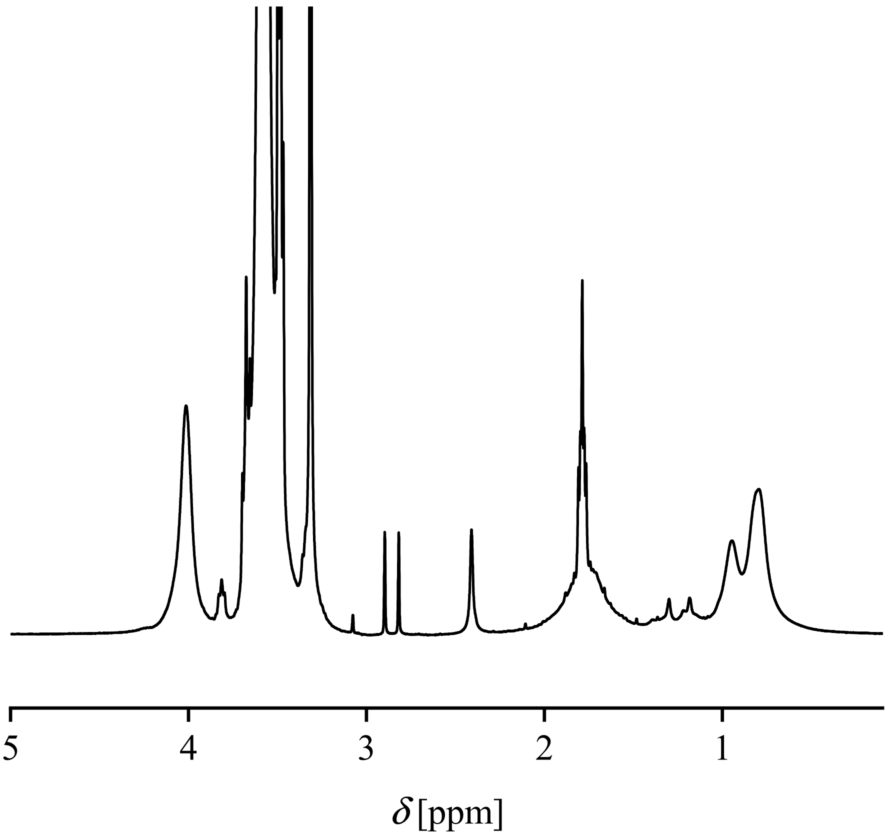

Figure S4: ^{1}H NMR spectrum of polymer **P1** (300 MHz, $CDCl_3$).

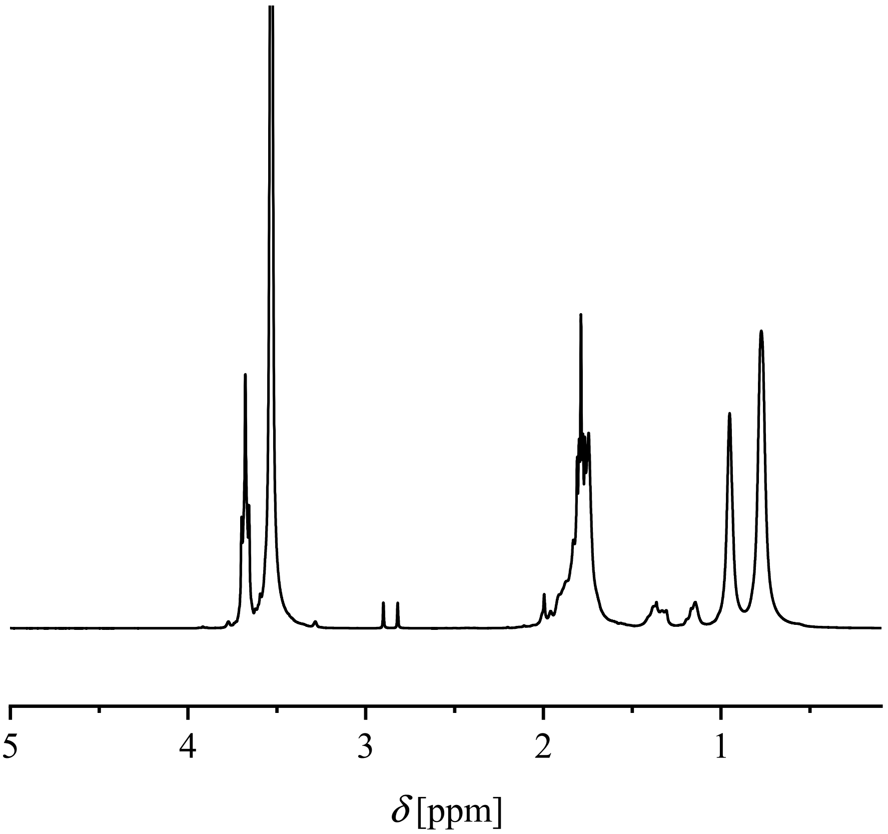

Figure S5: ^{1}H NMR spectrum of polymer **P2** (300 MHz, $CDCl_3$).

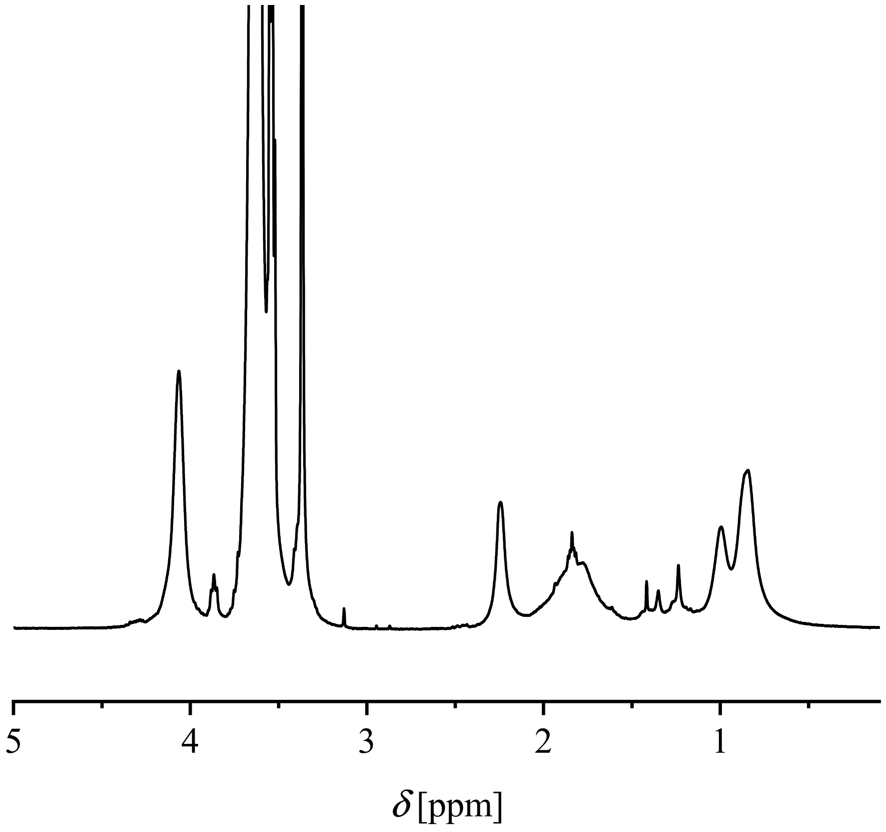

Figure S6: ^{1}H NMR spectrum of polymer **P3** (300 MHz, $CDCl_3$).

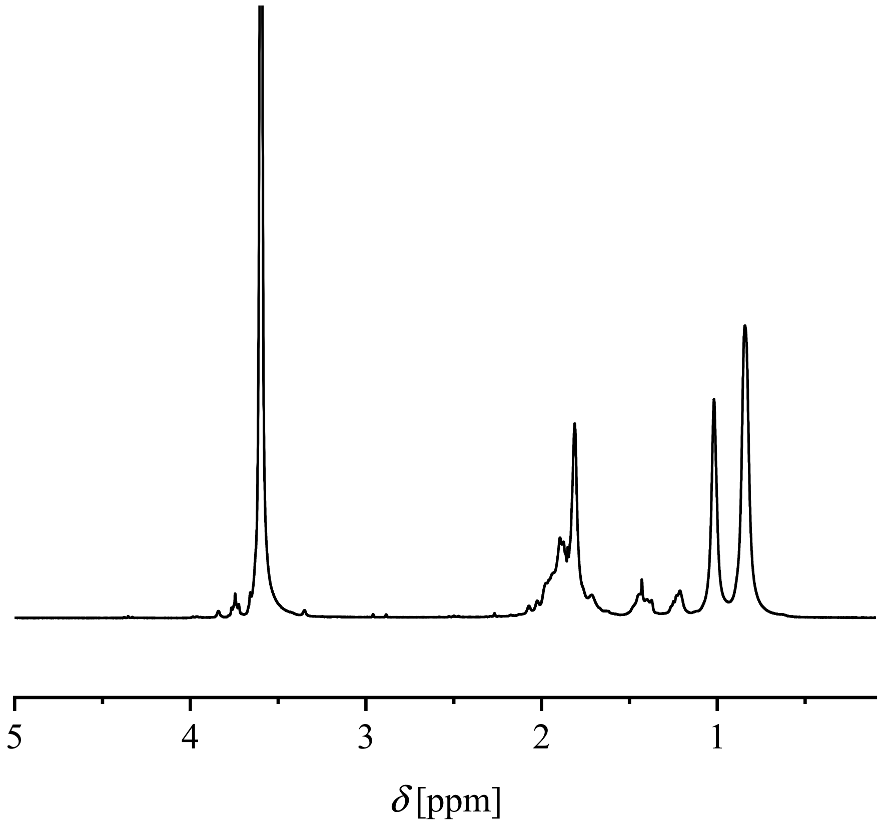

Figure S7: ^{1}H NMR spectrum of polymer **P4** (300 MHz, $CDCl_3$).

5. SEC-Diagrams

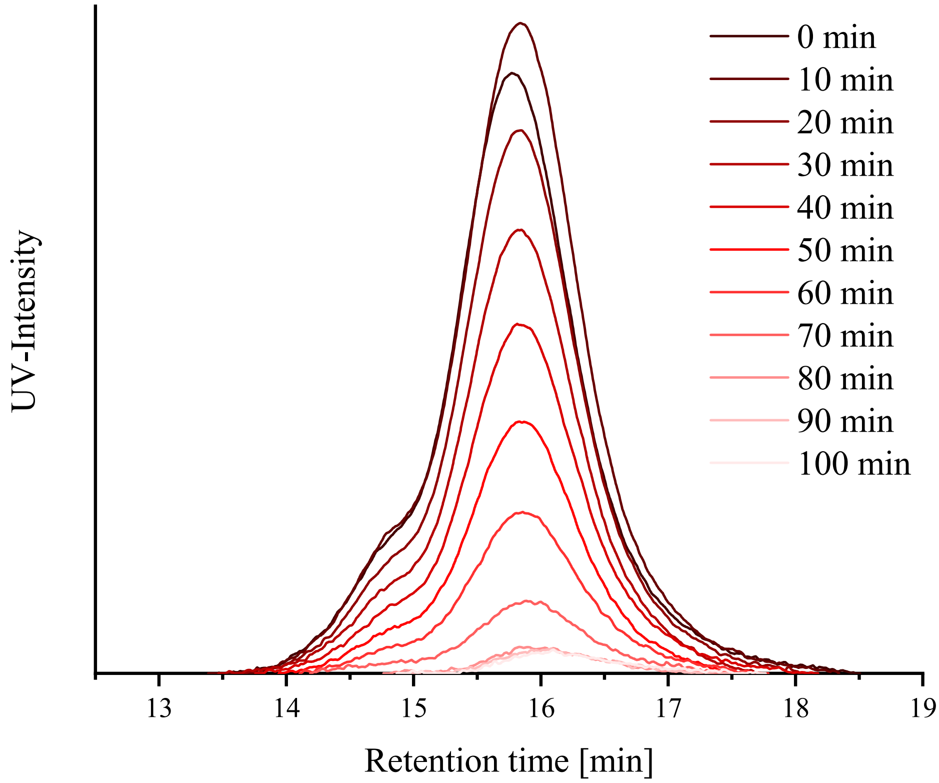

Figure S8: SEC-curves of the UV-induced degradation of poly(PEGMEMA) (**P1, E1**). Samples were taken every ten minutes for a period of 100 minutes (chloroform/isopropanol/triethylamine [94/2/4], PEG- and PMMA-standard, UV-detector (270 nm)).

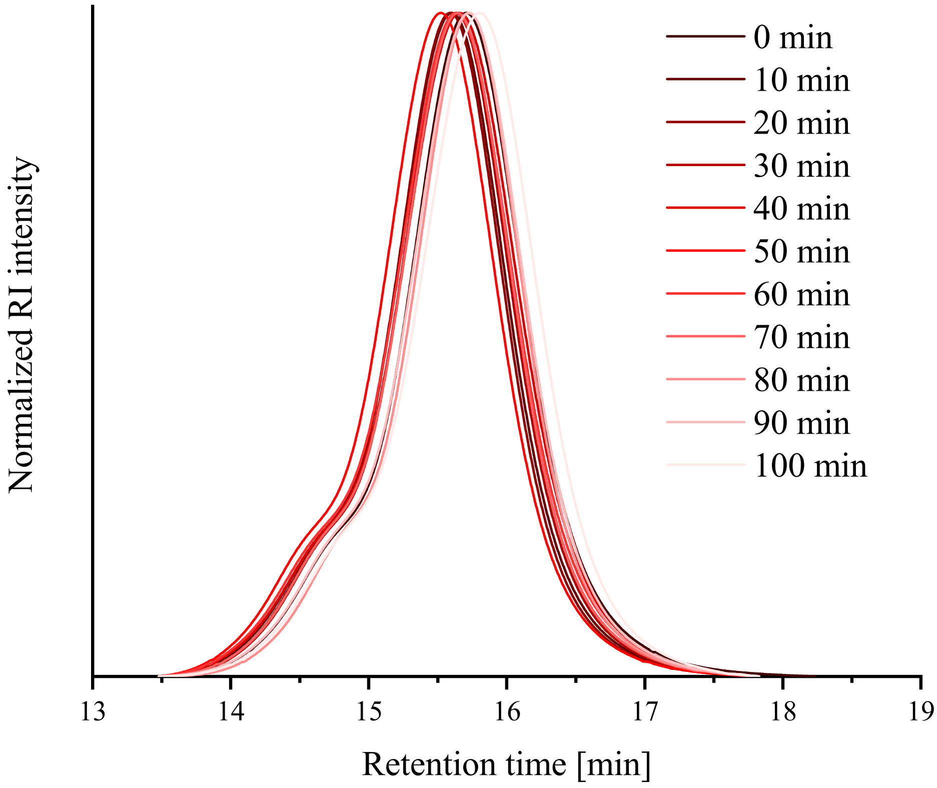

Figure S9: SEC-curves of the UV-induced degradation of poly(PEGMEMA) (**P1, E1**). Samples were taken every ten minutes for a period of 100 minutes (chloroform/isopropanol/triethylamine [94/2/4], PEG- and PMMA-standard, RI-detector).

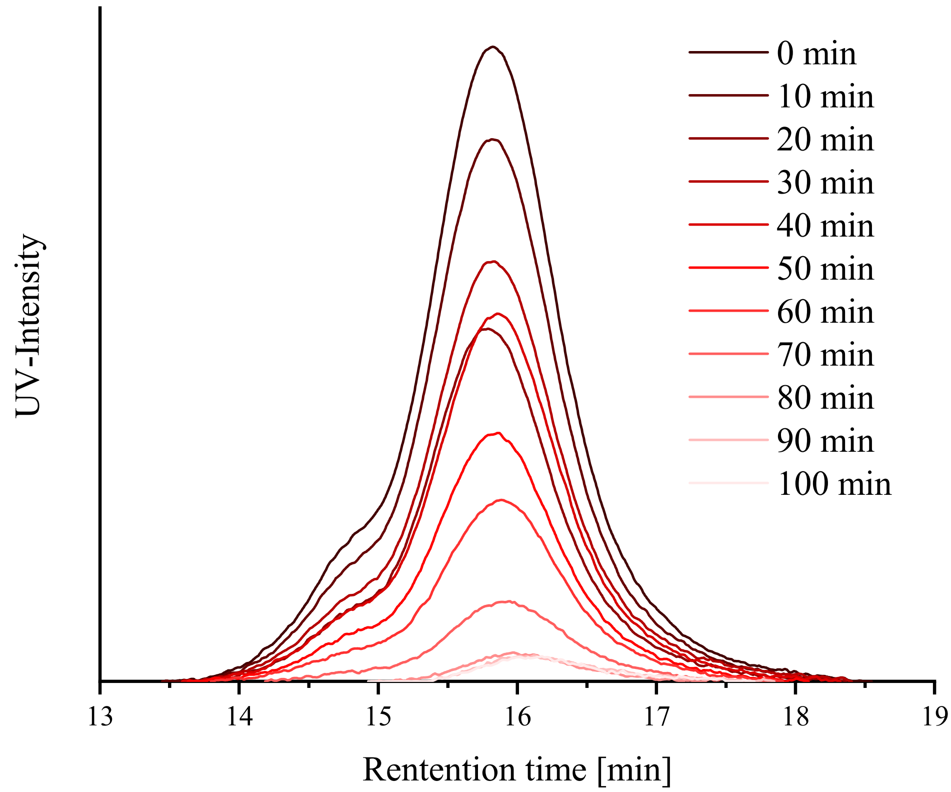

Figure S10: SEC-curves of the UV-induced degradation of poly(PEGMEMA) (**P1, E2**). Samples were taken every ten minutes for a period of 100 minutes (chloroform/isopropanol/triethylamine [94/2/4], PEG- and PMMA-standard, UV-detector (270 nm)).

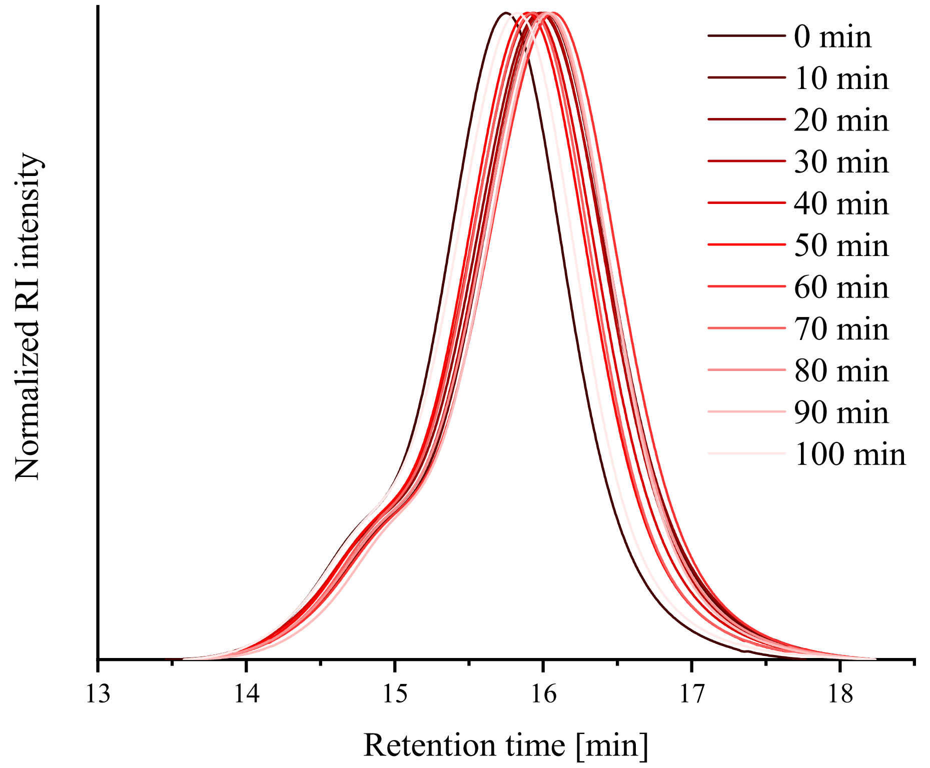

Figure S11: SEC-curves of the UV-induced degradation of poly(PEGMEMA) (**P1, E2**). Samples were taken every ten minutes for a period of 100 minutes (chloroform/isopropanol/triethylamine [94/2/4], PEG- and PMMA-standard, RI-detector).

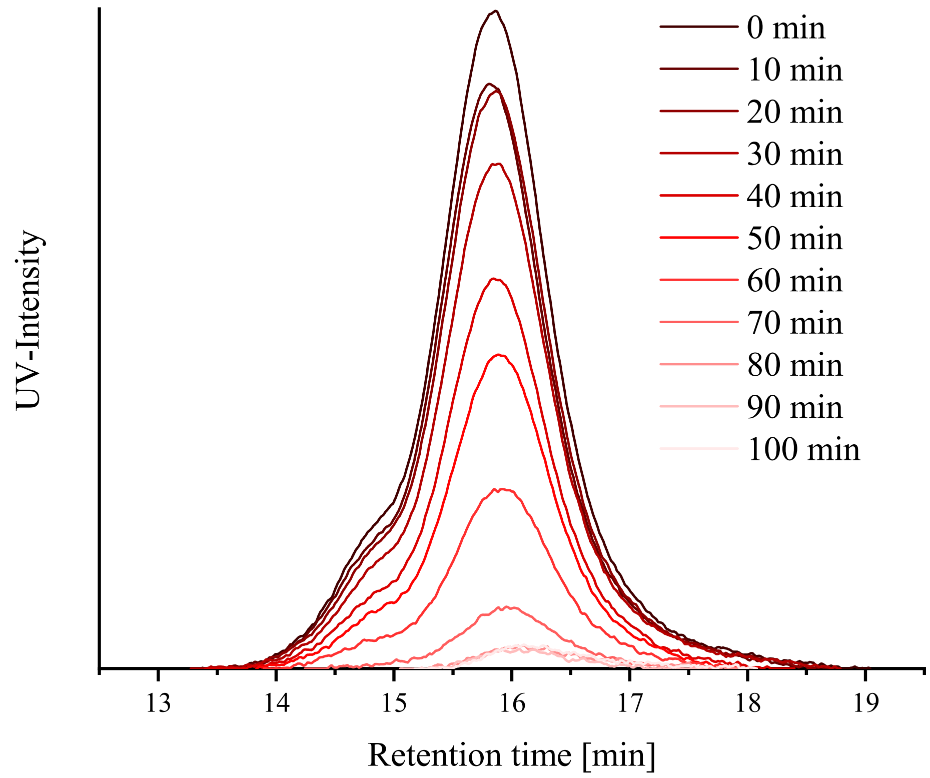

Figure S12: SEC-curves of the UV-induced degradation of poly(PEGMEMA) (**P1, E3**). Samples were taken every ten minutes for a period of 100 minutes (chloroform/isopropanol/triethylamine [94/2/4], PEG- and PMMA-standard, UV-detector (270 nm)).

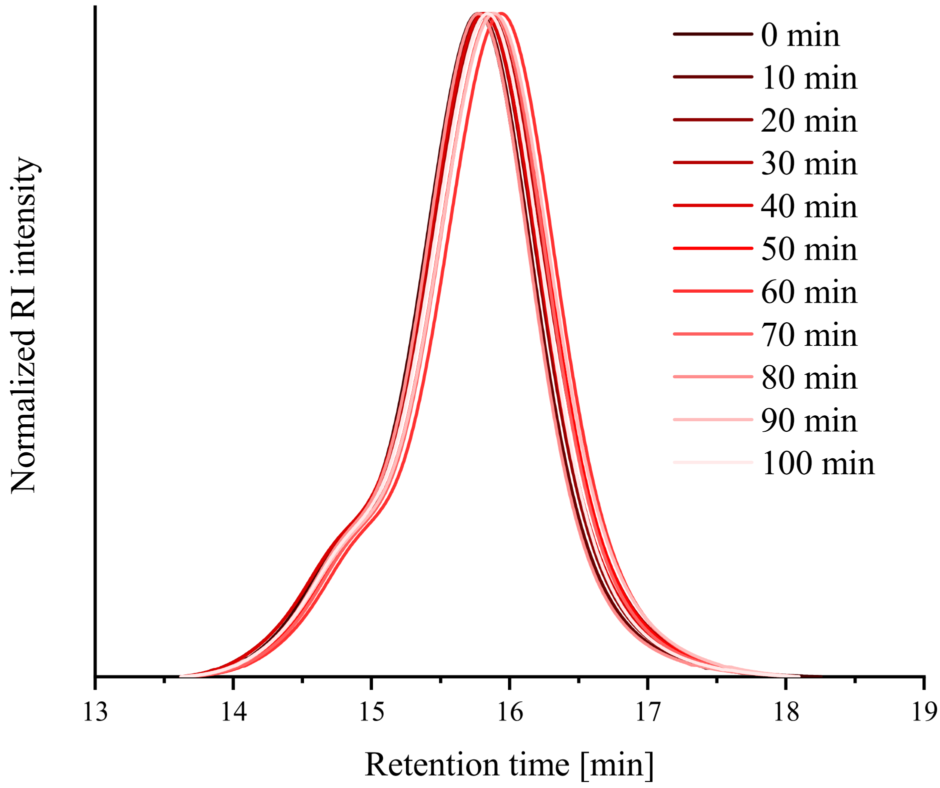

Figure S13: SEC-curves of the UV-induced degradation of poly(PEGMEMA) (**P1, E3**). Samples were taken every ten minutes for a period of 100 minutes (chloroform/isopropanol/triethylamine [94/2/4], PEG- and PMMA-standard, RI-detector).

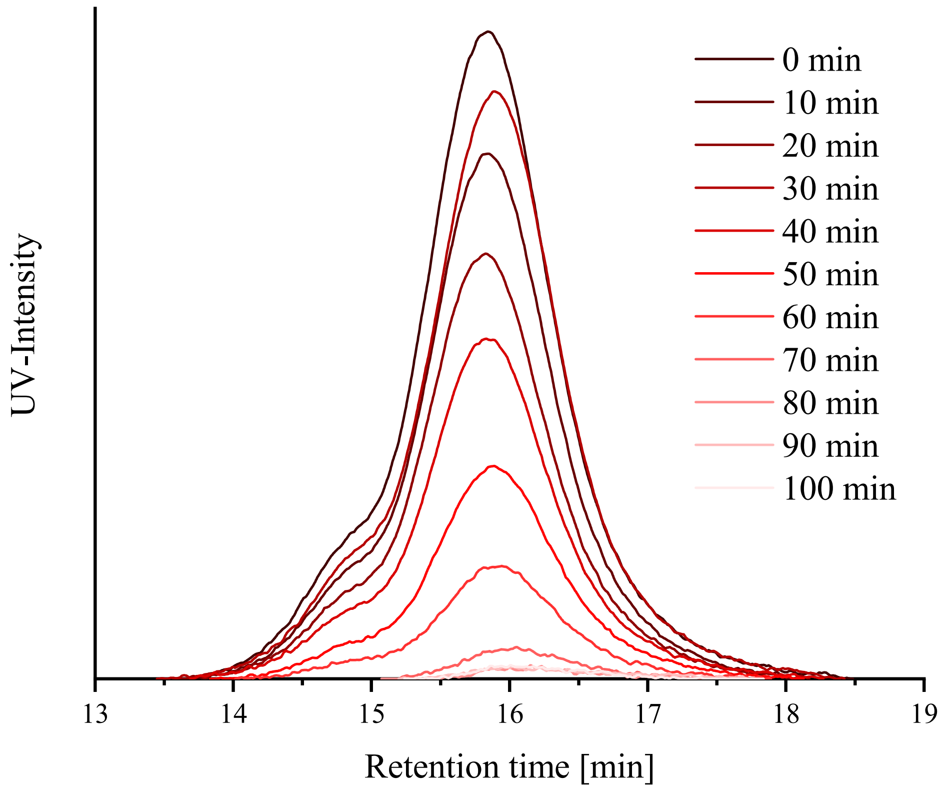

Figure S14: SEC-curves of the UV-induced degradation of poly(PEGMEMA) (**P1, E4**). Samples were taken every ten minutes for a period of 100 minutes (chloroform/isopropanol/triethylamine [94/2/4], PEG- and PMMA-standard, UV-detector (270 nm)).

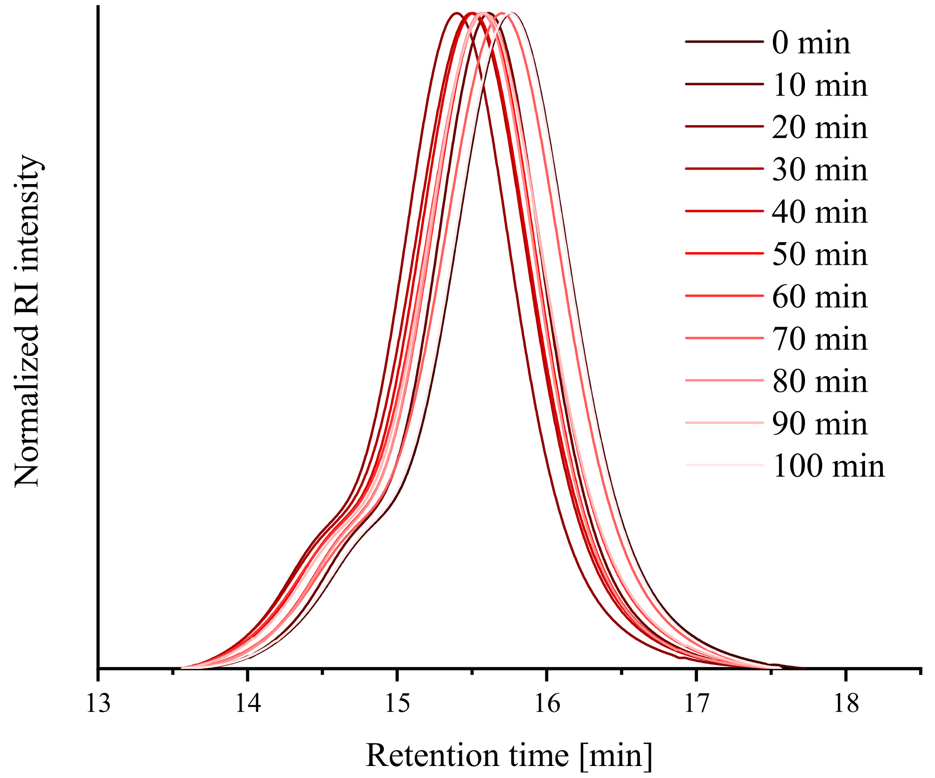

Figure S15: SEC-curves of the UV-induced degradation of poly(PEGMEMA) (**P1, E4**). Samples were taken every ten minutes for a period of 100 minutes (chloroform/isopropanol/triethylamine [94/2/4], PEG- and PMMA-standard, RI-detector).

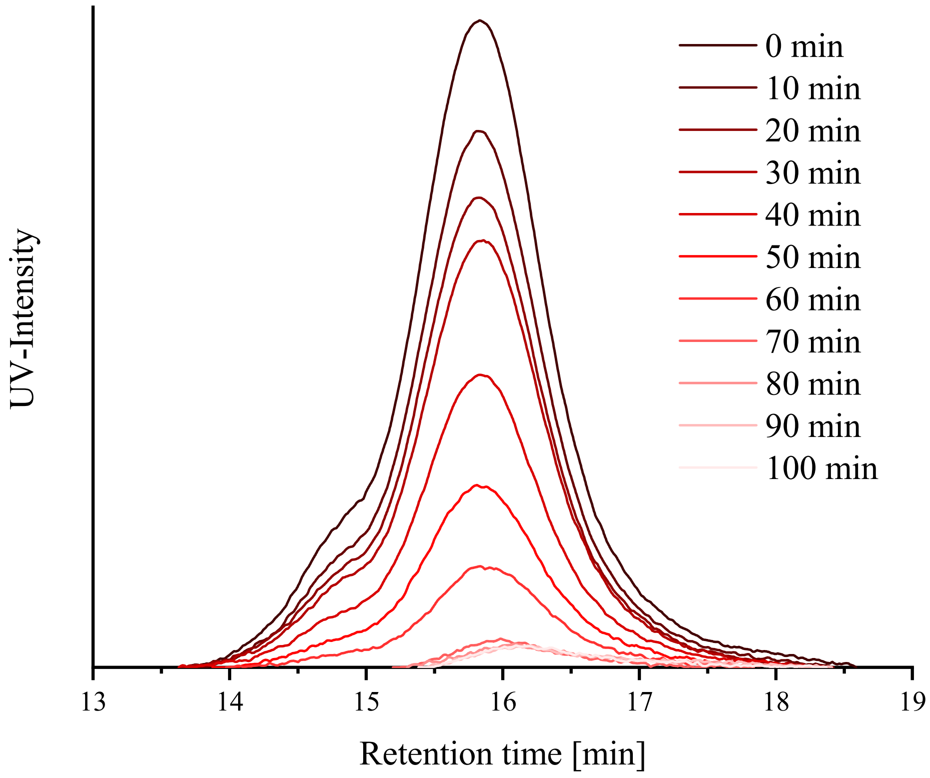

Figure S16: SEC-curves of the UV-induced degradation of poly(PEGMEMA) (**P1, E5**). Samples were taken every ten minutes for a period of 100 minutes (chloroform/isopropanol/triethylamine [94/2/4], PEG- and PMMA-standard, UV-detector (270 nm)).

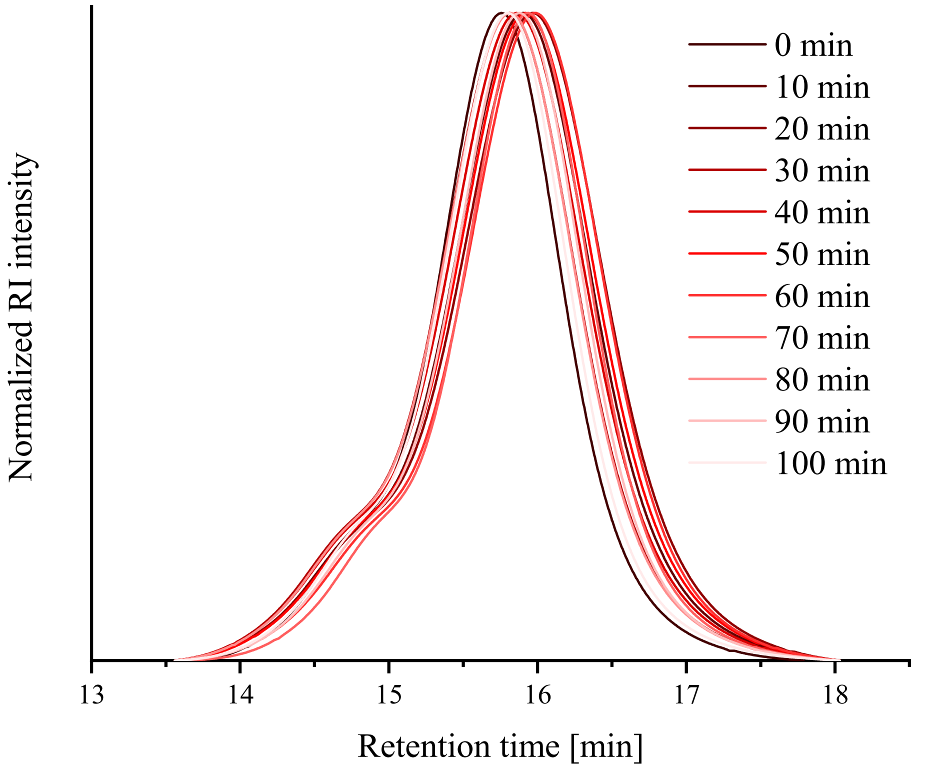

Figure S17: SEC-curves of the UV-induced degradation of poly(PEGMEMA) (**P1, E5**). Samples were taken every ten minutes for a period of 100 minutes (chloroform/isopropanol/triethylamine [94/2/4], PEG- and PMMA-standard, RI-detector).

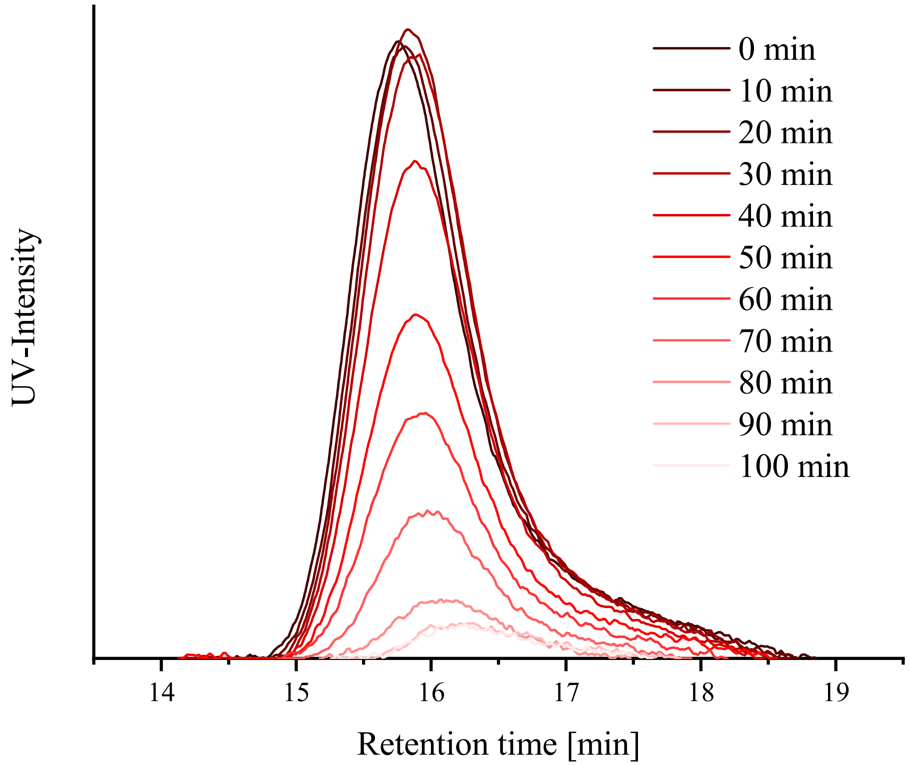

Figure S18: SEC-curves of the UV-induced degradation of PMMA (**P2, E6**). Samples were taken every ten minutes for a period of 100 minutes (chloroform/isopropanol/triethylamine [94/2/4], PMMA-standard, UV-detector (270 nm)).

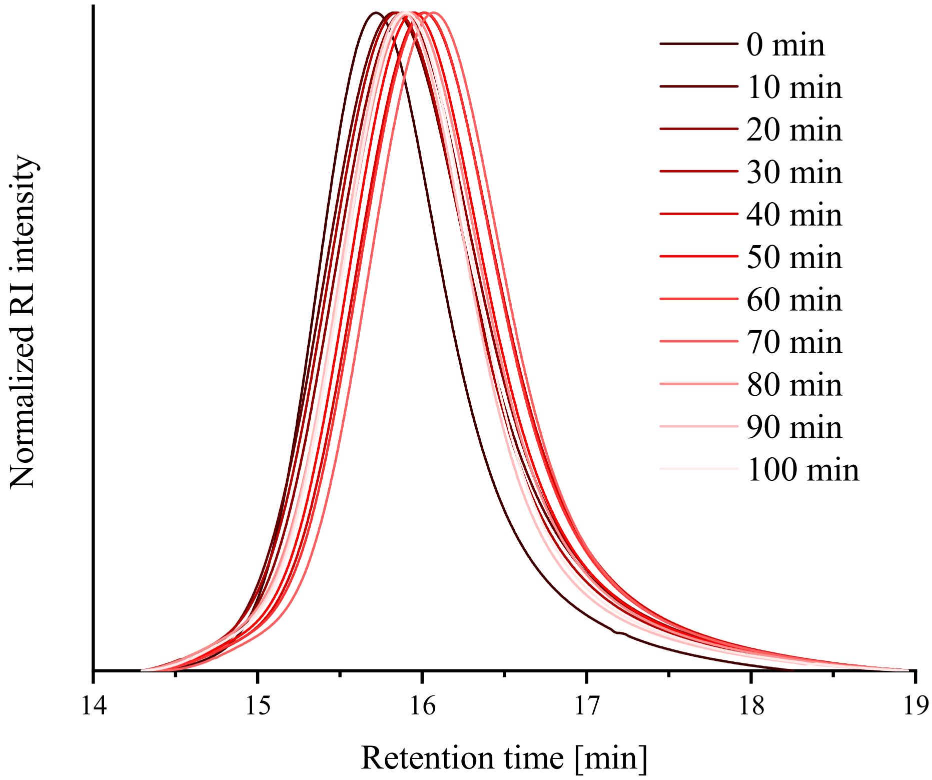

Figure S19: SEC-curves of the UV-induced degradation of PMMA (**P2, E6**). Samples were taken every ten minutes for a period of 100 minutes (chloroform/isopropanol/triethylamine [94/2/4], PMMA-standard, RI-detector).

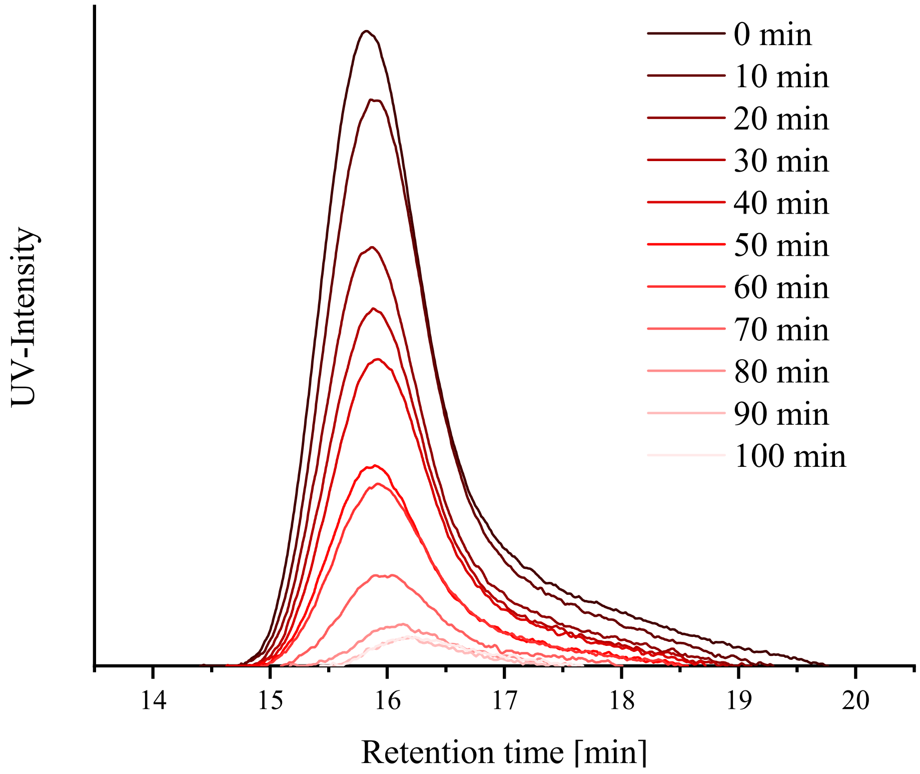

Figure S20: SEC-curves of the UV-induced degradation of PMMA (**P2, E7**). Samples were taken every ten minutes for a period of 100 minutes (chloroform/isopropanol/triethylamine [94/2/4], PMMA-standard, UV-detector (270 nm)).

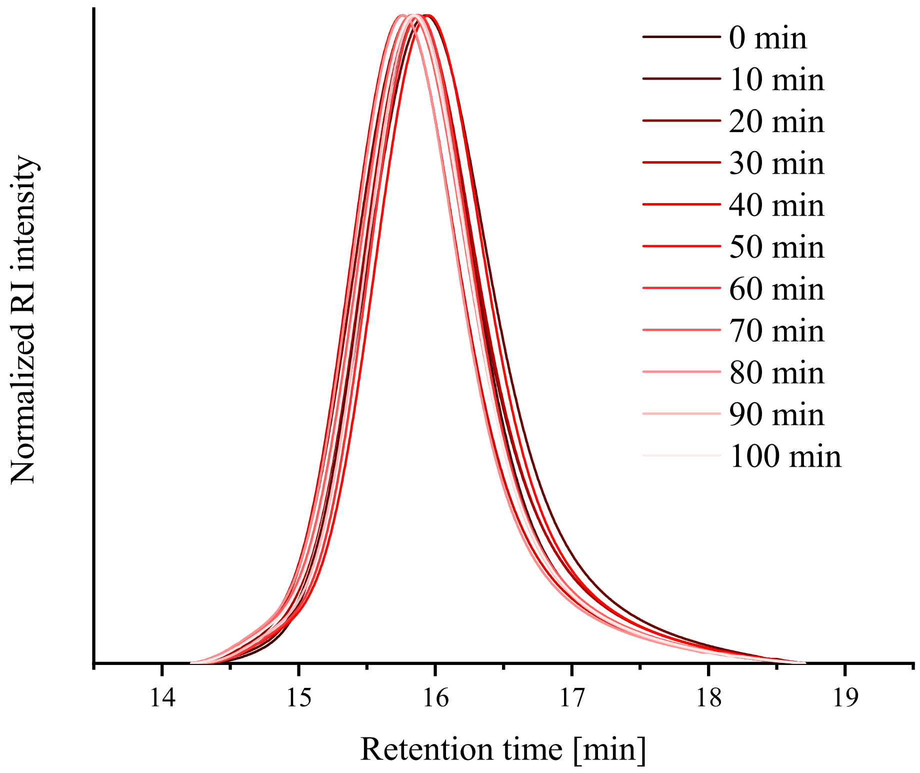

Figure S21: SEC-curves of the UV-induced degradation of PMMA (**P2, E7**). Samples were taken every ten minutes for a period of 100 minutes (chloroform/isopropanol/triethylamine [94/2/4], PMMA-standard, RI-detector).

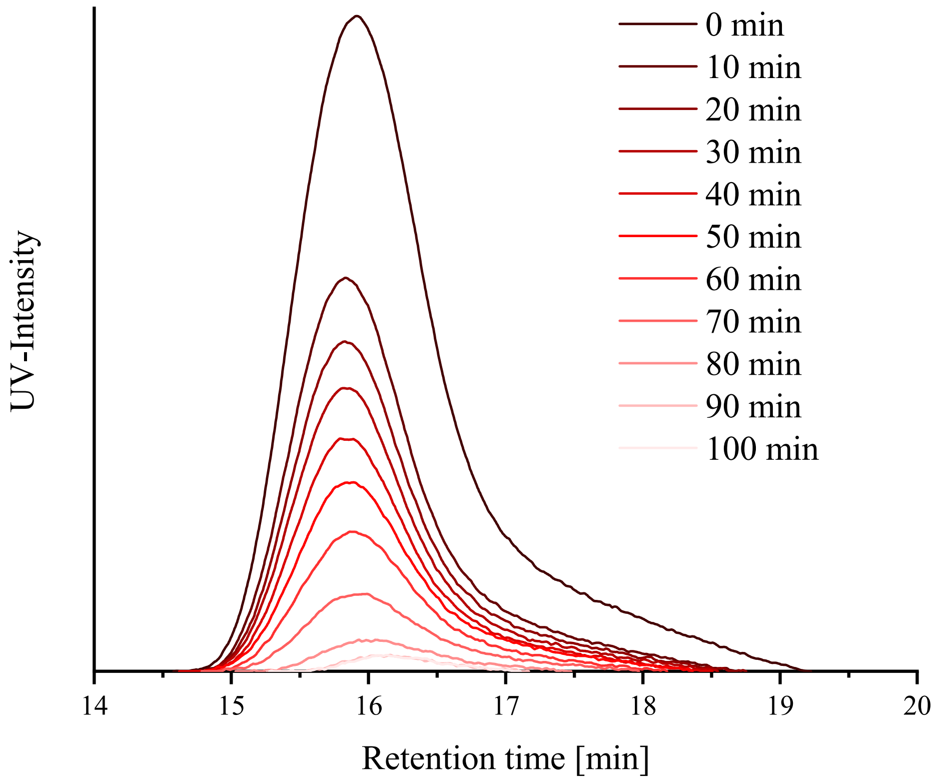

Figure S22: SEC-curves of the UV-induced degradation of PMMA (**P2, E8**). Samples were taken every ten minutes for a period of 100 minutes (chloroform/isopropanol/triethylamine [94/2/4], PMMA-standard, UV-detector (270 nm)).

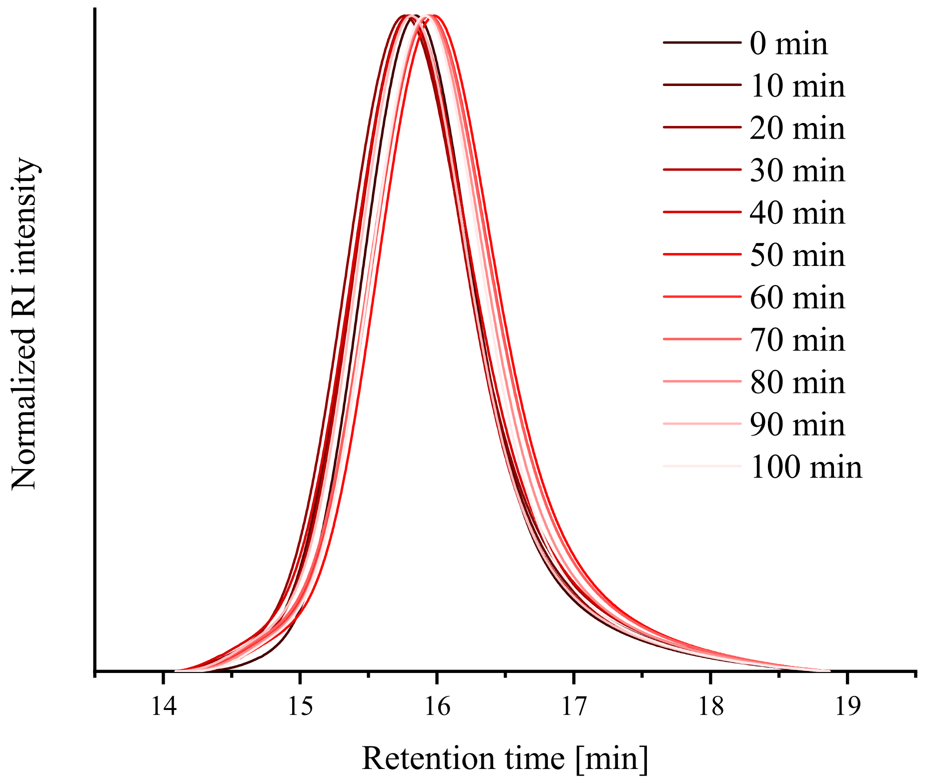

Figure S23: SEC-curves of the UV-induced degradation of PMMA (**P2, E8**). Samples were taken every ten minutes for a period of 100 minutes (chloroform/isopropanol/triethylamine [94/2/4], PMMA-standard, RI-detector).

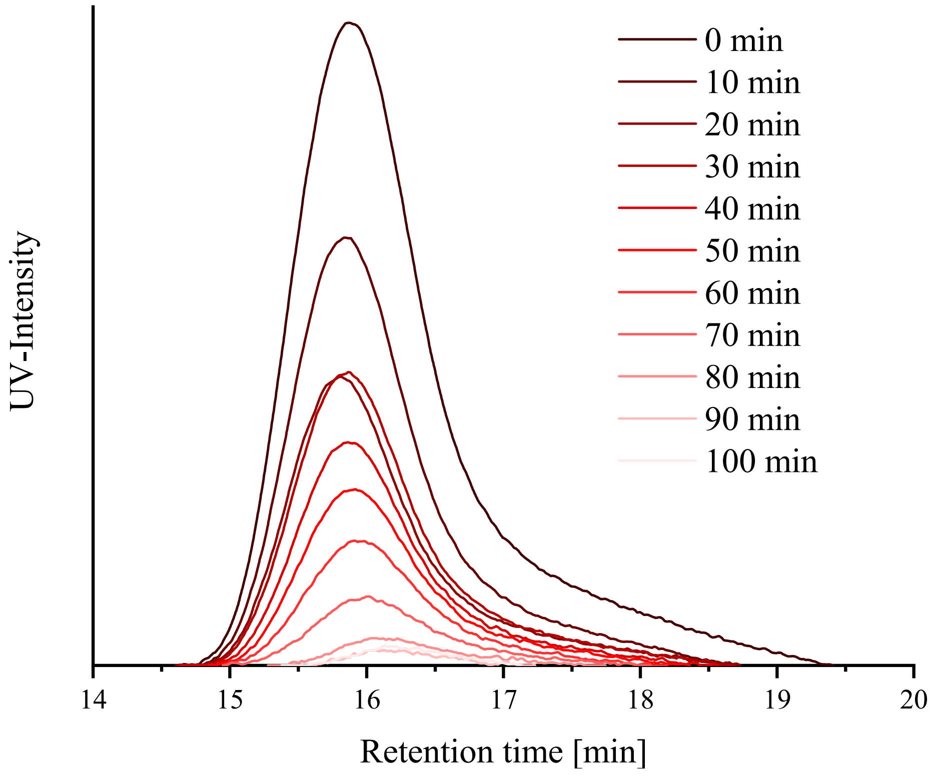

Figure S24: SEC-curves of the UV-induced degradation of PMMA (**P2, E9**). Samples were taken every ten minutes for a period of 100 minutes (chloroform/isopropanol/triethylamine [94/2/4], PMMA-standard, UV-detector (270 nm)).

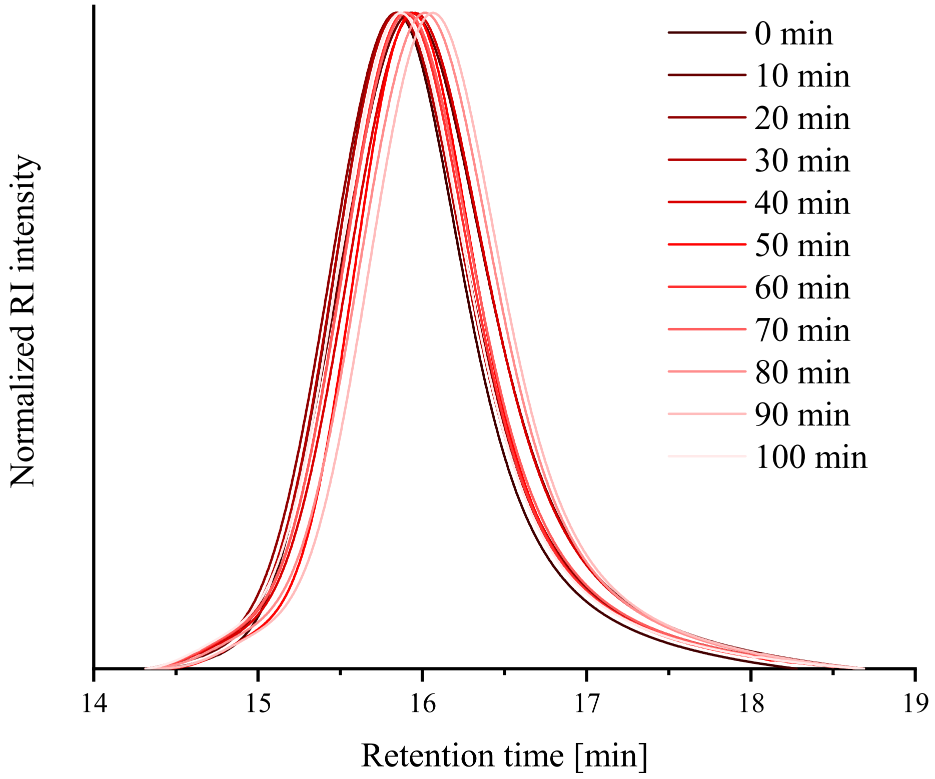

Figure S25: SEC-curves of the UV-induced degradation of PMMA (**P2, E9**). Samples were taken every ten minutes for a period of 100 minutes (chloroform/isopropanol/triethylamine [94/2/4], PMMA-standard, RI-detector).

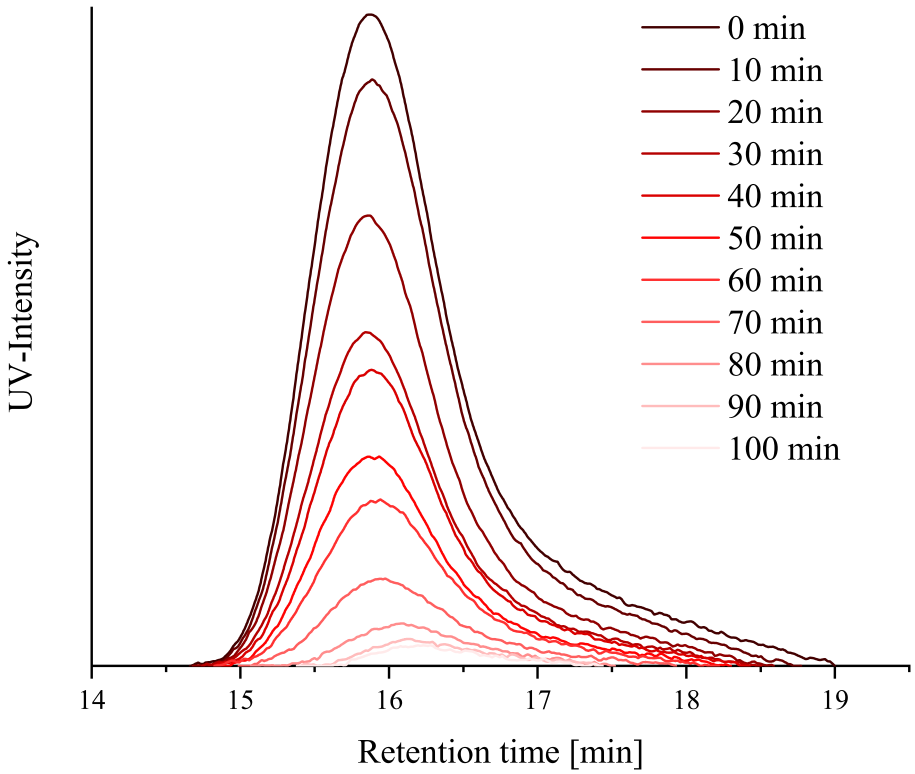

Figure S26: SEC-curves of the UV-induced degradation of PMMA (**P2, E10**). Samples were taken every ten minutes for a period of 100 minutes (chloroform/isopropanol/triethylamine [94/2/4], PMMA-standard, UV-detector (270 nm)).

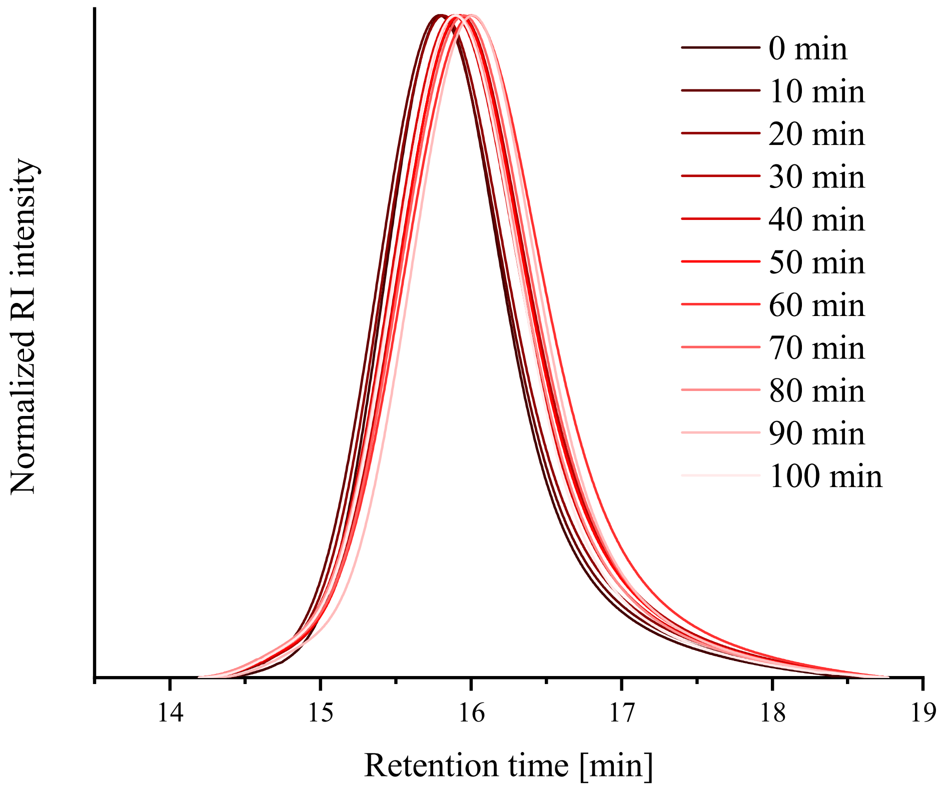

Figure 27: SEC-curves of the UV-induced degradation of PMMA (**P2, E10**). Samples were taken every ten minutes for a period of 100 minutes (chloroform/isopropanol/triethylamine [94/2/4], PMMA-standard, RI-detector).

6. SEC-Data

Table S2: Average molar mass and dispersity of each sampling within experiment **E1**. Values obtained by SEC measurements using a RI-detector (chloroform/isopropanol/triethylamine [94/2/4], PEG-standard).

Exp.	Time [min]	M_n [g mol^{-1}]	M_w [g mol^{-1}]	Đ
	0	11,900	14,700	1.2
	10	11,500	13,900	1.21
	20	11,700	14,100	1.21
	30	11,600	14,000	1.21
	40	11,500	13,900	1.21
**E1**	50	11,500	13,800	1.21
	60	11,500	13,900	1.21
	70	11,400	13,800	1.21
	80	11,400	13,700	1.21
	90	11,300	13,600	1.21
	100	11,300	13,600	1.21

Table S3: Average molar mass and dispersity of each sampling within experiment **E1**. Values obtained by SEC measurements using a RI-detector (chloroform/isopropanol/triethylamine [94/2/4], PMMA-standard).

Exp.	Time [min]	M_n [g mol^{-1}]	M_w [g mol^{-1}]	Đ
	0	22,600	26,800	1.19
	10	22,000	26,100	1.18
	20	22,300	26,300	1.18
	30	22,200	26,200	1.18
	40	22,000	25,900	1.18
**E1**	50	22,000	25,900	1.17
	60	22,100	25,600	1.16
	70	22,300	26,000	1.16
	80	22,400	26,000	1.16
	90	22,200	25,800	1.16
	100	21,900	25,700	1.17

Table S4: Average molar mass and dispersity of each sampling within experiment **E2**. Values obtained by SEC measurements using a RI-detector (chloroform/isopropanol/triethylamine [94/2/4], PEG-standard).

Exp.	Time [min]	M_n [g mol^{-1}]	M_w [g mol^{-1}]	Đ
	0	11,700	14,000	1.2
	10	11,700	14,100	1.2
	20	11,900	14,300	1.2
	30	11,700	14,100	1.2
	40	11,600	13,900	1.2
**E2**	50	11,700	14,100	1.21
	60	11,500	13,900	1.21
	70	11,500	13,900	1.21
	80	11,500	13,900	1.21
	90	11,300	13,700	1.22
	100	11,100	13,600	1.22

Table S5: Average molar mass and dispersity of each sampling within experiment **E2**. Values obtained by SEC measurements using a RI-detector (chloroform/isopropanol/triethylamine [94/2/4], PMMA-standard).

Exp.	Time [min]	M_n [g mol^{-1}]	M_w [g mol^{-1}]	Đ
	0	22,100	26,200	1.19
	10	22,200	26,300	1.19
	20	22,800	26,800	1.18
	30	22,200	26,400	1.19
	40	22,000	26,100	1.19
**E2**	50	22,400	26,400	1.18
	60	22,000	25,800	1.18
	70	22,200	26,000	1.17
	80	22,200	26,200	1.18
	90	22,000	25,900	1.17
	100	21,600	25,600	1.19

Table S6: Average molar mass and dispersity of each sampling within experiment **E3**. Values obtained by SEC measurements using a RI-detector (chloroform/isopropanol/triethylamine [94/2/4], PEG-standard).

Exp.	Time [min]	M_n [g mol^{-1}]	M_w [g mol^{-1}]	Đ
	0	11,400	13,700	1.21
	10	11,600	14,000	1.21
	20	11,400	13,800	1.21
	30	11,400	13,700	1.2
	40	11,400	13,700	1.21
**E3**	50	11,200	13,600	1.21
	60	11,200	13,600	1.21
	70	11,200	13,600	1.21
	80	11,200	13,600	1.21
	90	11,400	13,700	1.2
	100	10,900	13,300	1.21

Table S7: Average molar mass and dispersity of each sampling within experiment **E3**. Values obtained by SEC measurements using a RI-detector (chloroform/isopropanol/triethylamine [94/2/4], PMMA-standard).

Exp.	Time [min]	M_n [g mol^{-1}]	M_w [g mol^{-1}]	Đ
	0	21,800	25,800	1.18
	10	22,300	26,200	1.17
	20	22,100	25,900	1.17
	30	21,900	25,800	1.18
	40	21,900	25,800	1.18
**E3**	50	21,700	25,500	1.18
	60	21,600	25,400	1.18
	70	21,700	25,500	1.17
	80	21,800	25,700	1.18
	90	21,700	25,700	1.18
	100	21,200	25,000	1.18

Table S8: Average molar mass and dispersity of each sampling within experiment **E4**. Values obtained by SEC measurements using a RI-detector (chloroform/isopropanol/triethylamine [94/2/4], PEG-standard).

Exp.	Time [min]	M_n [g mol^{-1}]	M_w [g mol^{-1}]	Đ
	0	11,500	13,800	1.2
	10	11,400	13,700	1.2
	20	11,500	13,800	1.2
	30	11,100	13,300	1.2
	40	11,500	13,700	1.2
**E4**	50	11,200	13,500	1.2
	60	11,200	13,400	1.19
	70	11,300	13,500	1.19
	80	11,400	13,800	1.2
	90	11,700	14,000	1.19
	100	11,700	14,000	1.19

Table S9: Average molar mass and dispersity of each sampling within experiment **E4**. Values obtained by SEC measurements using a RI-detector (chloroform/isopropanol/triethylamine [94/2/4], PMMA-standard).

Exp.	Time [min]	M_n [g mol^{-1}]	M_w [g mol^{-1}]	Đ
	0	22,000	25,900	1.17
	10	21,900	25,800	1.18
	20	22,200	26,000	1.17
	30	21,300	25,100	1.18
	40	22,000	25,700	1.17
**E4**	50	21,600	25,400	1.18
	60	21,600	25,400	1.17
	70	21,800	25,500	1.17
	80	22,000	25,800	1.17
	90	22,600	26,300	1.17
	100	22,400	26,200	1.17

Table S10: Average molar mass and dispersity of each sampling within experiment **E5**. Values obtained by SEC measurements using a RI-detector (chloroform/isopropanol/triethylamine [94/2/4], PEG-standard).

Exp.	Time [min]	M_n [g mol^{-1}]	M_w [g mol^{-1}]	Đ
	0	11,600	13,900	1.2
	10	11,700	14,000	1.19
	20	11,800	14,000	1.19
	30	11,600	13,900	1.2
	40	11,700	14,000	1.19
**E5**	50	11,800	14,100	1.19
	60	11,600	13,900	1.2
	70	11,500	13,900	1.21
	80	11,500	13,800	1.2
	90	11,400	13,700	1.21
	100	11,300	13,700	1.21

Table S11: Average molar mass and dispersity of each sampling within experiment **E5**. Values obtained by SEC measurements using a RI-detector (chloroform/isopropanol/triethylamine [94/2/4], PMMA-standard).

Exp.	Time [min]	M_n [g mol^{-1}]	M_w [g mol^{-1}]	Đ
	0	22,300	26,300	1.18
	10	22,300	26,200	1.18
	20	22,300	26,200	1.17
	30	22,200	26,100	1.18
	40	22,400	26,300	1.17
**E5**	50	22,500	26,400	1.17
	60	22,300	26,100	1.17
	70	22,200	26,200	1.18
	80	22,100	26,000	1.18
	90	22,000	25,800	1.18
	100	21,900	25,800	1.18

Table S12: Average molar mass and dispersity of each sampling within experiment **E6**. Values obtained by SEC measurements using a RI-detector (chloroform/isopropanol/triethylamine [94/2/4], PMMA-standard).

Exp.	Time [min]	M_n [g mol^{-1}]	M_w [g mol^{-1}]	Đ
	0	19,500	22,300	1.15
	10	18,600	21,600	1.15
	20	18,300	21,300	1.16
	30	17,900	21,000	1.17
	40	18,100	21,100	1.17
**E6**	50	17,800	21,000	1.18
	60	17,800	21,000	1.17
	70	17,500	20,600	1.18
	80	17,300	20,400	1.18
	90	16,900	20,300	1.2
	100	17,000	20,300	1.19

Table S13: Average molar mass and dispersity of each sampling within experiment **E7**. Values obtained by SEC measurements using a RI-detector (chloroform/isopropanol/triethylamine [94/2/4], PMMA-standard).

Exp.	Time [min]	M_n [g mol^{-1}]	M_w [g mol^{-1}]	Đ
	0	17,700	20,700	1.17
	10	19,000	21,700	1.14
	20	18,800	21,700	1.15
	30	18,700	21,700	1.16
	40	18,400	21,700	1.18
**E7**	50	18,600	21,700	1.17
	60	18,100	21,500	1.19
	70	18,300	21,600	1.18
	80	18,100	21,500	1.19
	90	18,100	21,500	1.19
	100	18,100	21,400	1.18

Table S14: Average molar mass and dispersity of each sampling within experiment **E8**. Values obtained by SEC measurements using a RI-detector (chloroform/isopropanol/triethylamine [94/2/4], PMMA-standard).

Exp.	Time [min]	M_n [g mol^{-1}]	M_w [g mol^{-1}]	Đ
	0	17,700	20,900	1.18
	10	18,300	21,400	1.17
	20	18,900	22,000	1.16
	30	18,100	21,400	1.18
	40	18,200	21,300	1.17
**E8**	50	18,200	21,200	1.17
	60	18,000	21,000	1.17
	70	18,000	21,100	1.17
	80	17,800	21,000	1.18
	90	17,800	21,100	1.19
	100	17,100	20,400	1.19

Table S15: Average molar mass and dispersity of each sampling within experiment **E9**. Values obtained by SEC measurements using a RI-detector (chloroform/isopropanol/triethylamine [94/2/4], PMMA-standard).

Exp.	Time [min]	M_n [g mol^{-1}]	M_w [g mol^{-1}]	Đ
	0	18,300	21,000	1.15
	10	18,200	20,900	1.15
	20	18,300	21,200	1.16
	30	18,200	21,300	1.17
	40	18,300	21,200	1.16
**E9**	50	17,900	21,300	1.19
	60	17,800	21,100	1.18
	70	18,100	21,300	1.17
	80	18,100	21,100	1.17
	90	18,000	21,100	1.17
	100	17,700	20,800	1.18

Table S16: Average molar mass and dispersity of each sampling within experiment **E10**. Values obtained by SEC measurements using a RI-detector (chloroform/isopropanol/triethylamine [94/2/4], PMMA-standard).

Exp.	Time [min]	M_n [g mol^{-1}]	M_w [g mol^{-1}]	Đ
	0	18,400	21,400	1.16
	10	17,700	20,800	1.17
	20	18,000	21,100	1.17
	30	18,100	21,000	1.16
	40	17,600	20,700	1.17
**E10**	50	18,300	21,400	1.17
	60	18,400	21,200	1.15
	70	18,300	21,200	1.16
	80	18,100	21,100	1.17
	90	18,000	20,100	1.17
	100	17,600	20,800	1.18

Table S17: Average values of the average molar mass, the respective standard deviation (SD) and dispersity of each sampling within the respective experiment type (**E1** to **E5** and **E6** to **E10**) Values obtained by SEC measurements using a RI-detector (chloroform/isopropanol/triethylamine [94/2/4], PEG/PMMA-standard).

Exp.	Time [min]	M_n [g mol^{-1}]	SD [g mol^{-1}]	M_w [g mol^{-1}]	SD [g mol^{-1}]	Đ
	0	11,600	192	14,000	396	1.20
	10	11,600	130	13,900	152	1.20
	20	11,700	207	14,000	212	1.20
	30	11,500	239	13,800	316	1.20
**E1 – E5**	40	11,500	114	13,800	134	1.20
**(PEG-**	50	11,500	277	13,800	277	1.20
**standard)**	60	11,400	187	13,700	230	1.20
	70	11,400	130	13,700	182	1.21
	80	11,400	122	13,800	114	1.21
	90	11,400	164	13,700	152	1.21
	100	11,300	297	13,600	251	1.21
	0	22160	305	26200	394	1.18
	10	22140	182	26120	192	1.18
	20	22340	270	26240	351	1.17
	30	21960	391	25920	507	1.18
**E1 – E5**	40	22060	195	25960	241	1.18
**(PMMA-**	50	22040	404	25920	476	1.18
**standard)**	60	21920	311	25660	297	1.17
	70	22040	270	25840	321	1.17
	80	22100	224	25940	195	1.17
	90	22100	332	25900	235	1.17
	100	21800	442	25660	434	1.18
	0	18,300	736	21,300	635	1.16
	10	18,400	483	21,300	409	1.16
	20	18,500	378	21,500	378	1.16
**E6 – E10**	30	18,200	300	21,300	295	1.17
**(PMMA-**	40	18,100	311	21,200	361	1.17
**standard)**	50	18,200	321	21,300	259	1.18
	60	18,000	249	21,200	207	1.17
	70	18,000	329	21,200	365	1.17

80	17,900	349	21,000	396	1.18
90	17,800	493	20,800	593	1.18
100	17,500	453	20,700	434	1.18
